# Examining trends in inequality in the use of reproductive health care services in Ghana and Nigeria

**DOI:** 10.1186/s12884-018-2102-9

**Published:** 2018-12-13

**Authors:** Oluwasegun Jko Ogundele, Milena Pavlova, Wim Groot

**Affiliations:** 1 Department of Health Services Research, CAPHRI, Maastricht University Medical Center, Faculty of Health, Medicine and Life Sciences, Maastricht University, PO Box 616, 6200MD Maastricht, The Netherlands; 2 Top Institute Evidence-Based Education Research (TIER), Maastricht University, Maastricht, The Netherlands

**Keywords:** Concentration index, Inequality, Inequity, Reproductive services, Ghana, Nigeria

## Abstract

**Background:**

Equitable use of reproductive health care services is of critical importance since it may affect women’s and children’s health. Policies to reduce inequality in access to reproductive health care services are often general and frequently benefit the richer population. This is known as the inverse equity situation. We analyzed the magnitude and trends in wealth-related inequalities in the use of family planning, antenatal and delivery care services in Ghana and Nigeria. We also investigate horizontal inequalities in the determinants of reproductive health care service use over the years.

**Methods:**

We use data from Ghana’s (2003, 2008 and 2014) and Nigeria’s (2003, 2008 and 2013) Demographic and Health Surveys. We use concentration curves and concentration indices to measure the magnitude of socioeconomic-related inequalities and horizontal inequality in the use of reproductive health care services.

**Results:**

Exposure to family planning information via mass media, antenatal care at private facilities are more often used by women in wealthier households. Health worker’s assistance during pregnancy outside a facility, antenatal care at government facilities, childbirth at home are more prevalent among women in poor households in both Ghana and Nigeria. Caesarean section is unequally spread to the disadvantage of women in poorer households in Ghana and Nigeria. In Nigeria, women in wealthier households have considerably more unmet needs for family planning than in Ghana. Country inequality was persistent over time and women in poorer households in Nigeria experienced changes that are more inequitable over the years.

**Conclusion:**

We observe horizontal inequalities among women who use reproductive health care. These inequalities did not reduce substantially over the years. The gains made in reducing inequality in use of reproductive health care services are short-lived and erode over time, usually before the poorest population group can benefit. To reduce inequality in reproductive health care use, interventions should not only be pro-poor oriented, but they should also be sustainable and user-centered.

**Electronic supplementary material:**

The online version of this article (10.1186/s12884-018-2102-9) contains supplementary material, which is available to authorized users.

## Background

Equitable provision of reproductive health care services is of critical importance since it affects, among others, individual and economic development and bears on universally recognized human rights. The loss of healthy life years due to morbidity or mortality resulting from reproductive ill-health among pregnant women is highest in Sub-Saharan Africa [1]. This increases poverty and impedes the economic growth of nations since it impacts on child development and women’s labor force participation [2–4]. In Sub-Saharan Africa countries, reproductive health care services are not affordable to everyone in need, leading to unequal access to care [5–7].

Policies to reduce inequality in access to reproductive health care services, particularly in Sub-Saharan African countries, often have unintended and unwanted consequences. Such as health providers preference for urban and educated clients and the language barrier between provider and client [8]. Likewise, due to the inefficient distribution of health resources, policies often fail the poorest population, inadvertently widening the poor-rich gap [9]. This is often referred to as the inverse equity hypothesis [10]. Evidence on the relationship between government policies and access to services suggests that service delivery usually undermine benefits to the poor. For example, public health spending even though adequate can be allocated inefficiently and further precipitate between-group inequalities [11, 12]. Other studies have confirmed that access to healthcare innovations can be unequally distributed and to the advantage of richer households creating stratification in favor of the higher socioeconomic groups [13–19]. Pre-sustainable development goals era, research showed that interventions that address family planning, as well as maternal health care, are inequitable [20].

In light of this, low- and middle-income countries have implemented pro-poor initiatives with the goal to advance equitable access to quality reproductive health-care services. One example is the reproductive care services information channels in Nigeria [21]. Another is the provision of insurance schemes and community-based health programs in Ghana [22, 23]. Some of such successful interventions have been scaled-up, however with non-replicable successes [24, 25]. Both countries have put in place different health promotion schemes to attain a common goal of reducing inequities associated with the delivery of reproductive health care service such as the fee exemption for maternity care in Ghana [26, 27] and the national health insurance scheme in Nigeria [28]. This study contributes to the literature by investigating the underlying mechanisms of inverse equity for subsequent initiatives for underserved populations [10]. Ghana and Nigeria, through the sustainable development agenda, have agreed to foster equitable access to reproductive health care services [29].

The study analyzes the magnitude and trends in wealth-related inequality in the use of reproductive health care services (family planning and maternal care) in Ghana and Nigeria and provides insight into horizontal inequalities by describing the changes in the determinants of inequalities in the access to reproductive health care services over the years. An assessment of equity changes is essential to establish if policies addressing socioeconomic inequality improve the use of care.

In 2003, the fee exemption for maternity care commenced in four regions of Ghana (The Central, Northern, Upper West, and Upper East Regions), chosen due to the high poverty and maternal mortality levels and the low levels of supervised deliveries [26]. This policy was expanded in 2005 to cover the other six regions of Ghana. Thus all pregnant women in Ghana are exempted from payments for maternity care services such as prenatal visits, childbirth care (physiological childbirth and childbirth with medical assistance), caesarian section, and postnatal visit in all facilities [27]. Health insurance is compulsory for formal sector workers and voluntary for informal-sector workers and is reported to cover 65% of the population [30, 31]. The insurance premiums vary geographically and are ambiguously based on ability to pay with no clear guideline to determine premium levels [32]. However, significant differences in the use of maternity care persist [32–36]. In a concurrent effort to promote access to health care, reproductive care services included, the community-based health planning services in Ghana provide community-level services targeted at poor mothers and provide services including family planning, supervising delivery and maternity care [25]. The community-based health planning services have been introduced to all districts/regions to facilitate access, especially for the population living further away from health care services.

Nigeria’s national health insurance scheme, initiated in 1999 and kicked off in 2005, is a social health insurance scheme aimed at improving access to health care and reducing associated cost. It was piloted in six regions among civil servants and formal sector employees, targeting 5 percent of the population [28]. Coverage through the NHIS remains less than 5% of the Nigerian population (NHIS, 2011). To broaden coverage, the community-based health insurance scheme, flagged off in 2008, was made available to the general population and subsidized for households, particularly in rural communities. The scheme is organized by community members and covers family planning services, antenatal care, as well as vaginal childbirth [37]. The community-based health insurance scheme allows for differences in premium rates, enrolment, and uptake of varied sexual and reproductive health care services across the country [37, 38]. Access to reproductive health care services in Nigeria remains underdeveloped and a large proportion of the population has no health coverage living most of the health expenditure to be borne by households.

The midwives service scheme was implemented in 2009 throughout the country as part of efforts to reach the rural communities and facilitate the adoption of skilled care among the populations by improving the capacity of public primary health facilities [38]. Allocation of midwives service scheme facilities is determined using geographic location as the factor with northeast and northwest regions emerging as a top priority, in part due to high maternal mortality rate and low access to services [24]. Though deemed to be making developments in implementation, highlighted setbacks include the non-availability of qualified midwives and retention of midwives [39]. Reports of horizontal variation in the use of reproductive health in the achievements of the midwives service scheme were reported [24].

Nigeria and Ghana were selected based on their governments having introduced a national health insurance program and other health promotion programs to address the inaccessibility of reproductive health care services [21, 25]. The introductions of these programs have met with different success and have contributed to differences in access and use of reproductive health care services between the two countries. The differences and similarities in the progress towards equality will provide information on constraints to access to services among the poor and improve policies that address these. Research on the equity effect of reproductive health care policies is limited.

## Methods

### Data

We used secondary data from the Demographic and Health Surveys (DHS). The surveys are conducted under an international program implemented by ICF International and funded by the USAID with contributions from UNICEF, UNFPA, WHO, and UNAIDS [40]. The DHS are cross-sectional and nationally representative surveys in low- and middle-income countries. The DHS adopt a multi-stage cluster design and samples selected for enumeration are ensured to be representative and comparative across countries. The DHS involves a two-stage cluster and systematic sampling design with households selected at random. In both countries, the sampling accounted for differences in population distribution regionally as well as for the urban-rural spread. Designated households were enumerated without allowance for a change or replacement to prevent bias. Respondents were selected on the basis of being female (for the female survey) or male (for the male survey), aged 15-49 and whether the respondent was a usual member of the household or having spent the night prior the survey in the household. These surveys employed standard Demographic Health Surveys (DHS) questionnaires and techniques for data collection [40]. All eligible women aged 15–49 were interviewed with the Women’s Questionnaire. Eligible women are all women aged 15–49 who stayed in a selected household the night before the interview, irrespective of whether they were usual residents in the household or not. The Women’s Questionnaire was used to collect respondent’s individual characteristics including age, marital status, occupation, residence as well as other information on topics including; reproductive history; contraceptive knowledge and use; antenatal, delivery and postnatal care; marriage; attitudes about family planning.

Analyses were performed using data from the women’s response file, from the full DHS dataset of Ghana (2003, 2008, and 2014) and Nigeria (2003, 2008, and 2013). We use data from women who have had at least one birth in the 5 years prior to the survey. A summary of indicators used and information on missing data is available in Additional file 1.

### Measurement

We consider family planning, antenatal care, and delivery care services as essential aspects of reproductive healthcare. The dependent variables to indicate exposure to or use of family planning, antenatal care, and delivery care services were grouped in similar themes based on the WHO recommendations [41]. Table 1 shows the definition of the indicators used in the intervention areas examined. Responses to questions on the use of similar reproductive health care services were aggregated to produce one outcome variable (see Additional file 1 for grouping description). This was done to capture the different types of health care used during pregnancy. All dependent variables are dichotomized, taking the value 1 when a woman answered “Yes” to the questions and “0” if otherwise. The dependent variable indicating a woman’s unmet need for family planning is coded as 1 when a woman answered “No” to the question if she wanted last birth and “0” if she answered “Yes”. Control variables include a woman’s age, marital status, occupation, location, and region of residence. Coding of the dependent and control variables used are indicated in Additional file 1.

**Table 1:** Definition of indicators by intervention area used for the equity analysis

Indicators for family planning	
Family planning info: Health facility	Percentage of women told of family planning at a health facility
Family planning worker visit	Percentage of women who were visited by FP worker last 12 months
Family planning: TV	Percentage of women who heard family planning information on TV last months
Family planning: Print	Percentage of women who got family planning information on a newspaper last months
Modern contraceptive	Percentage of women who currently use by a modern method of contraceptive
Information on pregnancy complication	Percentage of women who were told about pregnancy complications
Family planning: unmet need	Percentage of women who wanted the last child later / wanted no more
Indicators for antenatal care	
Health worker’s (HW) assistance during pregnancy outside a facility	Percentage of pregnant women who had care at an informal setting
ANC: nurse assisted	Percentage of pregnant women who got assistance from a nurse/midwife during pregnancy
ANC: government health facility	Percentage of pregnant women who received antenatal care at a form of government/public health care center
ANC: Private health facility	Percentage of pregnant women who received antenatal care at a form the private healthcare center
ANC: 1st trimester	Percentage of pregnant women who received antenatal care in the first 12 weeks of pregnancy
ANC: 4+ tetanus injection	Percentage of pregnant women who received tetanus injections before birth
ANC: Home	Percentage of pregnant women who had antenatal care at a home
Indicators for delivery care	
Delivery: home	Percentage of pregnant women who had childbirth at a home
Delivery: government health facility	Percentage of pregnant women who had childbirth at a form of government/public health care center
Delivery: private health facility	Percentage of pregnant women who had childbirth at a form of a private health care center
Birth assistance: Doctor	Percentage of pregnant women who had childbirth assisted by a Doctor
Caesarean section	Percentage of pregnant women who had Caesarean section childbirth

#### Household Wealth

To measure household wealth, asset ownership and living conditions available in each DHS dataset were used. Wealth was measured by ownership of some or all consumer items and residence characteristics including electricity, radio, television, refrigerator, bicycle, motorcycle, car or truck, non-mobile phone, water source, type of toilet facility, flooring, wall, and roofing materials. These were used to create a wealth index score by adopting D Filmer and LH Pritchett [42] principal component analysis approach to generate the indicator weights for the household assets and subsequently weighted scores for all assets that were summed to create a household wealth index. The types of assets owned were similar between Ghana and Nigeria, though local context implied differences in the consumer items and residence characteristics used in the index. The asset includes weights that varied between countries. This has been described as the local perception of wealth approach.

### Equity analysis

To measure household wealth inequality in access to family planning, antenatal care, and delivery care services in Ghana and Nigeria over time, we use the concentration curve and associated index. The concentration curve plots the cumulative percentage of use of various health care services on the vertical axis (*y*-axis) against the cumulative percentage of women ranked by their household wealth on the horizontal axis (*x*-axis), beginning with the poorest and ending with the richest households. The equality line runs diagonally across the figure when women, irrespective of economic status have the same access to health care service, that is, all values on the *x*-axis equals all values on the *y*-axis [43]. A curve that lies below the equality line indicates that access to the health care service is concentrated among wealthier households. If the curve lies above the line of equality it implies the presence of inequity, that is, use of the health care service is concentrated among poorer households.

Concentration indices were used to assess the magnitudes and trend of horizontal inequity. Analyses were performed to measure absolute inequality in reproductive healthcare use. Concentration index (CI) range from -1.0 to +1.0; negative values of the CI indicate that the use of reproductive health care services is concentrated in poor households, positive values indicate among wealthy households, and 0 indicates the absence of household-wealth related inequality [43, 44]. For computation, a more convenient formula for the concentration index defines it in terms of the covariance between the healthcare outcome and the fractional rank in the household wealth distribution.

1 $$ \mathrm{CI}=\kern0.5em \frac{2}{\upmu}\ \mathit{\operatorname{cov}}\ \left(h,r\right) $$

where *h* is the healthcare outcome of interest, μ is the mean of *h* and *r* is the fractional rank of an individual in the household wealth distribution. Additional analyses performed test the null hypothesis of equality across groups to measure horizontal inequality, which is the hypothesis that the index is the same within a group. Comparison of the concentration indices within socioeconomic groups, including age, marital status, maternal occupation, location (rural or urban), and region of residence, was done using the homogeneity test as provided by O O'Donnell, S O'Neill, T Van Ourti and B Walsh [45].

We used sampling weights for all statistical analysis. Data analyses were performed using STATA version 15.1.

## Results

**Fig. 1:**
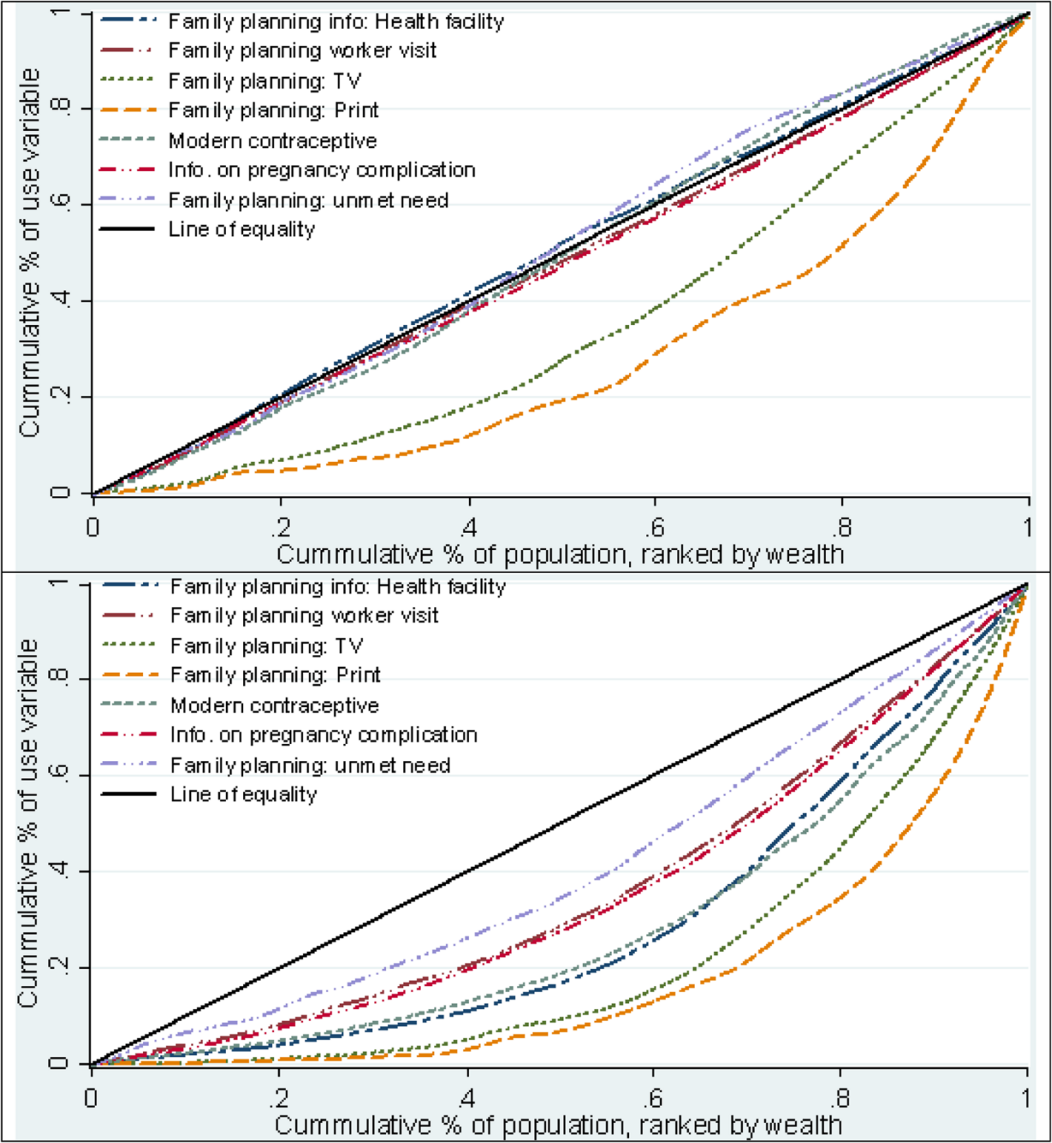
Concentration curves of use of family planning Ghana (2014) above and Nigeria (2013) below

**Fig. 2:**
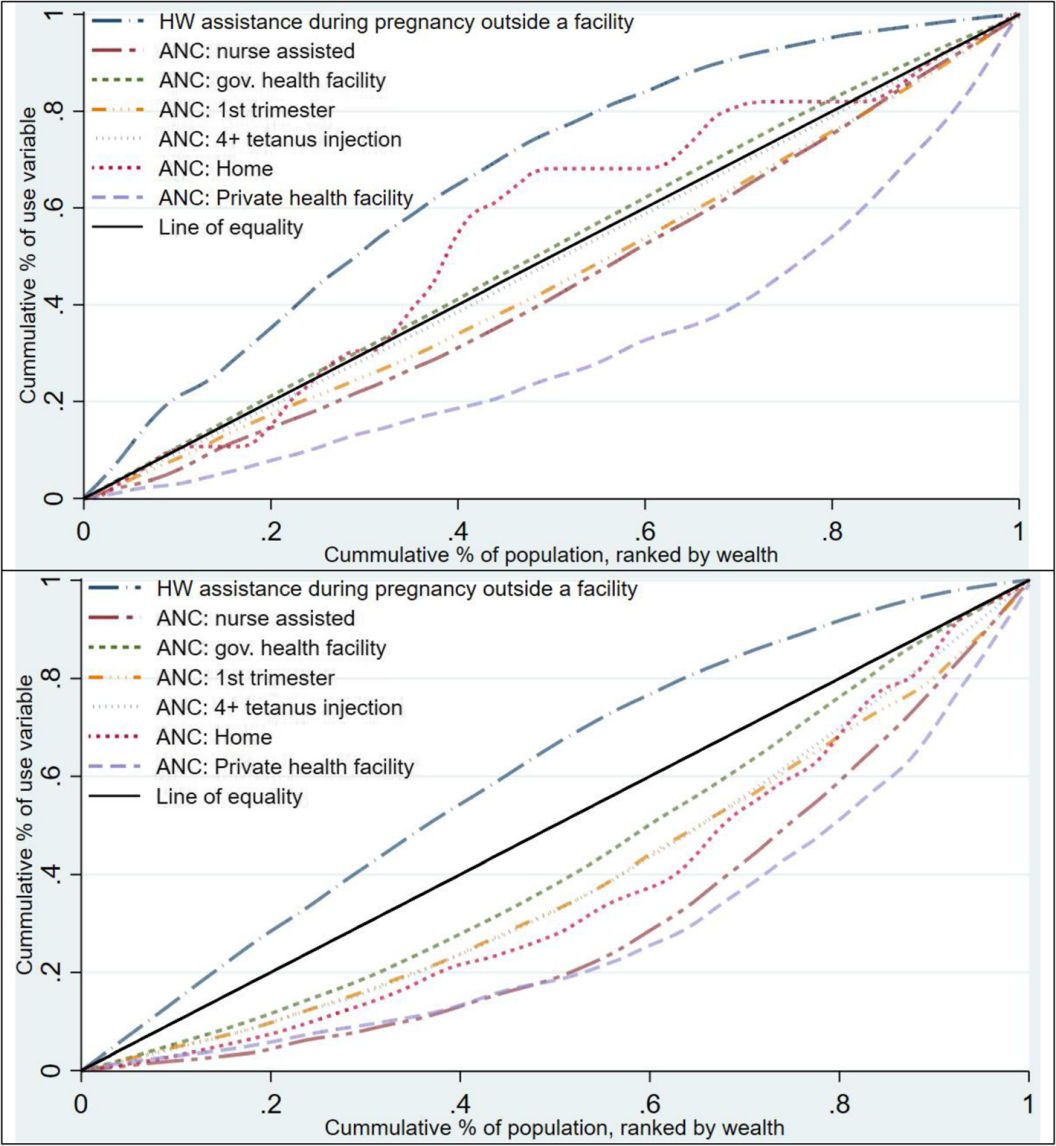
Concentration curves of use of antenatal care Ghana (2014) above and Nigeria (2013) below

**Fig. 3:**
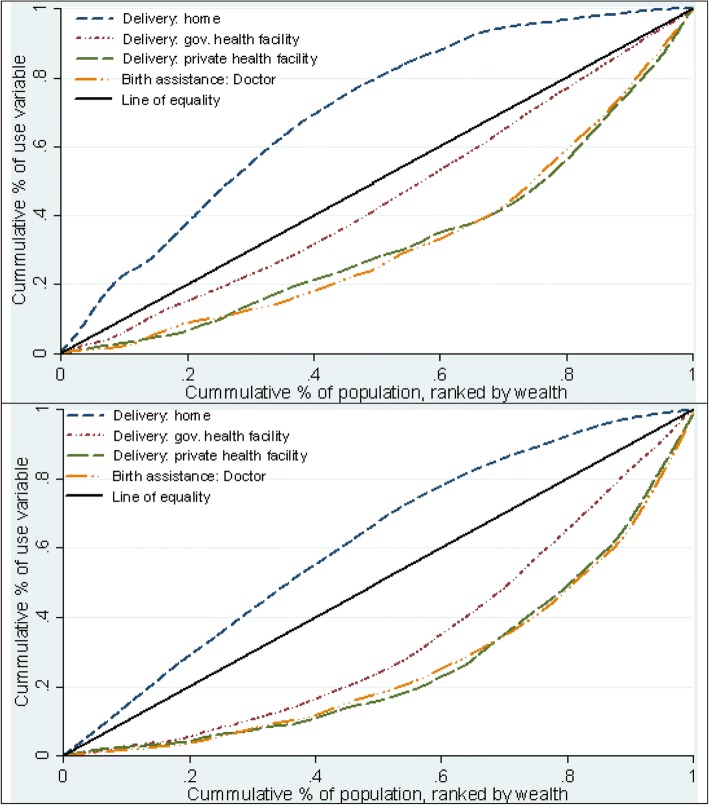
Concentration curves of use of delivery care Ghana (2014) above and Nigeria (2013) below

Figures 1, 2 and 3 show the concentration curves of reproductive health care service use. At the end of the observed years, it appears that reproductive health care services are being used less by women in Nigeria compared with Ghana. The distribution of the outcome variables in the poorest 20 percent, richest 20 percent and all women included in the analysis in Ghana and Nigeria (in Additional file 1) suggests changes in the proportion of women using reproductive services in both countries is irregular through the years. Additionally, the data suggest that the use of reproductive health services did not increase substantially among women in the poorest households.

### Concentration curve of household wealth-related inequality in use of reproductive health care services

In Figure 1, the top image indicates that the curves of family planning information via print and TV lie distinctively furthest away from the line of equality over the observed years in Ghana, suggesting that these services are to the disadvantage of women in poor households. The bottom image of Fig. 1 shows that all the curves describing the use of family planning services including the use of modern contraceptive lie below the line of equality observed in Nigeria. Lastly, the curve of unmet needs for family planning lies below the equality line and increased over the periods observed in Nigeria unlike in Ghana where the curve lies closely to the equality line all through (Additional files 2 and 3: Figures S1 and S2).

Figure 2 shows that in Ghana (top image), the concentration curve of antenatal care at private hospitals is below and furthest away from the equality line. Figure 2 (bottom image) suggests that all the examined antenatal care services are distributed to the disadvantage of women in poor households in Nigeria since the curve lies below the equity line. Other concentration curves depicting a woman’s use of antenatal care lie close to the equality line (Additional files 4 and 5: Figures S3 and S4).

Figures 3 (top and bottom images) show a clear picture of the curves for indicators associated with delivery care across the years in Ghana and Nigeria respectively. The curve of home births lies above the line of equality in both countries throughout the periods observed, indicating predominance among poor households (Additional files 6 and 7: Figures S5 and S6). Also, the curves depicting the use of private facility, Caesarean section, and assistance by a doctor during child delivery appears to follow a similar pattern and lie furthest away below the equity line throughout the years observed (Additional files 6 and 7: Figures S5 and S6).

### Concentration indices of household wealth-related inequality in the use of reproductive health care services

Table 2 shows that the value of the CI declined and remained positive for indicators of family planning information via TV ( +0.37 to +0.28), and print (+0.54 to +0.42) from 2003 to 2014 in Ghana. Examination of these indicators by maternal individual characteristics shows that CI values are positive and largest among women who are currently or previously married, agrarians, or live in the Upper East region of Ghana. Concentration indices for indicators of use of family planning information via TV or print medium are positive and high, above +0.43, throughout the years observed in Nigeria, indicating concentration among wealthier households. CI values for visits to health facilities or visits by family planning workers are negative between 2003 and 2014 in Ghana. Women who wanted to give birth no more or later indicated as unmet needs for family planning were insignificant across the years in Ghana. In Nigeria, CI values of unmet needs for family planning were positive and significant over the years. Individual estimates suggest that the degree of inequality associated with unmet needs for family planning is greatest among women in more wealthy households who are not working or in rural residence in Nigeria. Additional test results for group differences indicate that the magnitude of inequality of unmet needs for family planning is significantly different across occupation or residence types in the observed years.

**Table 2:** Concentration Indices with Covariates: Ghana (years 2003, 2008 & 2014) and Nigeria (years 2003, 2008 & 2013)

Service type / covariate	Ghana			Nigeria		
	2003	2008	2014	2003	2008	2013
Family planning info: health facility	-0.01	-0.04	-0.04*	0.14*	0.14*	0.15*
Age group						
15-24	0*	0.05	-0.02	0.14	0.18*	0.18*
25-49	-0.01	-0.06	-0.05*	0.13*	0.12*	0.14*
Marital status						
Never	-0.02	-0.06	-0.01	0.04	0.06	0.03
Currently / previously	-0.01	-0.04	-0.04*	0.14*	0.14*	0.16*
Maternal occupation						
Not working	0.05	0	0	0.2	0.18*	0.21*
Professional / sales	0	-0.04	-0.07*	0.14*	0.09*	0.12*
Agriculture	-0.01	-0.05	0	0.06	0.11*	0.08
Others	-0.02	0.02	-0.01	0.13	0.16*	0.14*
Location						
Urban	-0.02	-0.01	-0.02	0.02	0.08*	0.05*
Rural	-0.02	-0.01	-0.01	0.15	0.16*	0.19*
Family planning worker visit	-0.09*	-0.02	-0.05	0.25*	0.39*	0.4*
Age group						
15-24	0.01	0.05	0.01	0.22	0.49*	0.43*
25-49	-0.11*	-0.04	-0.06	0.25*	0.35*	0.38*
Marital status	-0.09*	-0.02	-0.05	0.25*	0.39*	0.4*
Never	-0.1	0.09	0.03	-0.03	-0.04	0.01
Currently / previously	-0.09*	-0.02	-0.06	0.26*	0.4*	0.41*
Maternal occupation						
Not working	0.07	0.15	-0.03	0.43*	0.51*	0.44*
Professional / sales	-0.18*	-0.08	0.02	0.27*	0.32*	0.37*
Agriculture	-0.06	-0.14*	0.02	0.07	0.25*	0.2*
Others	-0.15	0.06	-0.13	0.31	0.4*	0.36*
Location						
Urban	-0.15*	-0.01	0	0.18*	0.2*	0.14*
Rural	-0.04	-0.02	0	0.22	0.39*	0.42*
Family planning: TV	0.37*	0.4*	0.28*	0.5*	0.58*	0.56*
Age group						
15-24	0.3*	0.37*	0.26*	0.44*	0.6*	0.61*
25-49	0.39*	0.41*	0.29*	0.51*	0.56*	0.53*
Marital status						
Never	0.2*	0.22*	0.2*	0.12	0.29*	0.31*
Currently / previously	0.37*	0.42*	0.29*	0.51*	0.59*	0.57*
Maternal occupation						
Not working	0.33*	0.4*	0.24*	0.52*	0.64*	0.65*
Professional / sales	0.23*	0.27*	0.15*	0.45*	0.48*	0.5*
Agriculture	0.32*	0.46*	0.32*	0.51*	0.41*	0.32*
Others	0.27*	0.26*	0.21*	0.42*	0.51*	0.49*
Location						
Urban	0.17*	0.19*	0.13*	0.29*	0.28*	0.25*
Rural	0.32*	0.44*	0.35*	0.39*	0.63*	0.63*
Family planning: print	0.54*	0.52*	0.42*	0.43*	0.65*	0.64*
Age group						
15-24	0.5*	0.56*	0.25	0.4*	0.63*	0.62*
25-49	0.55*	0.51*	0.44*	0.44*	0.64*	0.62*
Marital status						
Never	0.28	0.5	0.36	0.22	0.36*	0.26
Currently / previously	0.55*	0.52*	0.43*	0.45*	0.66*	0.65*
Maternal occupation						
Not working	0.45*	0.46*	0.14	0.42*	0.7*	0.73*
Professional / sales	0.4*	0.43*	0.31*	0.4*	0.57*	0.56*
Agriculture	0.43*	0.55*	0.32	0.47	0.42*	0.5*
Others	0.43*	0.37*	0.19	0.38*	0.57*	0.59*
Location						
Urban	0.31*	0.36*	0.26*	0.34*	0.39*	0.36*
Rural	0.45*	0.54*	0.45*	0.3*	0.67*	0.67*
Modern contraceptive	0.01	-0.05	-0.06*	0.03	-0.04*	-0.02
Age group						
15-24	0.01	-0.05	-0.08*	0.06	0.02	0.02
25-49	0.01	-0.04	-0.05*	0.03	-0.05*	-0.02
Marital status						
Never	0.04	-0.17	-0.06	0.07	0.05	0.07*
Currently / previously	0.01	-0.04	-0.06*	0.03	-0.04*	-0.02
Maternal occupation						
Not working	-0.01	-0.08	-0.05	0.05	-0.05	-0.03
Professional / sales	0.02	-0.04	-0.08*	0.02	-0.03	-0.01
Agriculture	0.01	-0.07*	0	0.04	-0.09*	-0.03
Others	-0.02	-0.01	-0.07*	-0.02	0	-0.05
Location						
Urban	-0.02	-0.06	-0.07*	0.02	-0.02	0
Rural	0.02	-0.05	-0.02	0.03	-0.04*	-0.03
Information on pregnancy complication	0.08*	0.07*	0.02*	0.13*	0.12*	0.1*
Age group						
15-24	0.06*	0.06*	0	0.16*	0.12*	0.1*
25-49	0.09*	0.07*	0.02*	0.11*	0.11*	0.09*
Marital status						
Never	0.15*	0.1	0	0.1	0.1	0.08*
Currently / previously	0.08*	0.07*	0.02*	0.13*	0.12*	0.1*
Maternal occupation						
Not working	0.15*	0	0	0.17*	0.15*	0.1*
Professional / sales	0.06*	0.04*	0	0.11*	0.09*	0.09*
Agriculture	0.1	0.06*	0	0.1	0	0.07*
Others	0.1	0	0.03*	0.11*	0.11*	0.08*
Location						
Urban	0	0	0.02*	0.08*	0.07*	0.05*
Rural	0.07*	0.07*	0	0.09*	0.1*	0.08*
Family planning: unmet need	0.01	0.01	-0.02	0.05	0.18*	0.18*
Age group						
15-24	0.08*	0.06	0.05	0.11	0.25*	0.22*
25-49	-0.02	-0.01	-0.03	0.03	0.17*	0.17*
Marital status						
Never	-0.03	0.09*	-0.03	0.03	0.04	0
Currently / previously	0.01	-0.01	-0.02	0.05	0.19*	0.19*
Maternal occupation						
Not working	0.06	0.03	-0.04	0.08	0.21*	0.29*
Professional / sales	-0.04	-0.09*	-0.11*	0.07	0.16*	0.21*
Agriculture	0.07*	0.09*	0.08	0.06	0.12*	0.01
Others	-0.02	-0.06	-0.07	0.11	0.3*	0.19*
Location						
Urban	-0.02	-0.08	-0.11*	0.07	0.04	0.02
Rural	0.07*	0.07*	0.06	0.03	0.22*	0.21*

Note: Magnitudes of regional variation is available in Additional file 1

*p ≤ 0.01

Table 3 presents the values of the concentration indices of indicators of antenatal care services. Values show that the CI of health worker’s assistance during pregnancy outside a facility declined slightly from -0.25 in 2003, to -0.21 in 2008 and peaked at -0.27 in 2014 in Ghana. Comparison across groups show that the use of health worker’s assistance during pregnancy outside a facility was significantly different between occupation, residence, and region in Ghana for years 2003 and 2014; women in poor households in the professional / sales occupational category, urban residence, or live in Greater Accra had the greatest negative CI values. In Nigeria, for health worker’s assistance during pregnancy outside a facility, the values of CI indicate an increase from -0.17 in 2003 to -0.21 in 2008 and 2013. Test results show significant differences between age and occupational groups, residence type as well as the region of residence; women who are 25-49 years, professional / sales occupation, in urban residences, or live in the South East region of Nigeria consistently have the greatest negative CI values. For the observed years, CI values of antenatal care in government hospitals increased from -0.02 to -0.04 in Ghana, from -0.04 to -0.07 in Nigeria. Urban-rural differences among women became insignificant after 2003 in Ghana and significant from 2008 in Nigeria. A similar increase in CI values is observed for antenatal care at private hospitals from +0.24 to +0.36 in Ghana and +0.20 to +0.27 in Nigeria. Over the observed years, the CI magnitude of home antenatal care indicator declined and remained negative in Ghana. However, a change in Nigeria from negative to positive was noted, -0.23, -0.15 and +0.05. Results show that in the observed years, CI values increased for nurse assisted antenatal care in Nigeria, +0.33 to +0.39, while it reduced in Ghana, +0.29 to +0.11.

**Table 3:** Concentration Indices with Covariates: Ghana (years 2003, 2008 & 2014) and Nigeria (years 2003, 2008 & 2013)

Service type / covariate	Ghana			Nigeria		
	2003	2008	2014	2003	2008	2013
Health worker’s assistance during pregnancy outside a facility	-0.25*	-0.21*	-0.27*	-0.17*	-0.21*	-0.21*
Age group						
15-24	-0.23*	-0.06	-0.19*	-0.13*	-0.14*	-0.15*
25-49	-0.26*	-0.25*	-0.29*	-0.19*	-0.23*	-0.23*
Marital status						
Never	-0.21*	-0.2	-0.2	-0.23	-0.15*	-0.17*
Currently / previously	-0.25*	-0.21*	-0.28*	-0.17*	-0.21*	-0.21*
Maternal occupation						
Not working	-0.29*	-0.32*	-0.2*	-0.16*	-0.17*	-0.17*
Professional / sales	-0.35*	-0.2*	-0.29*	-0.19*	-0.28*	-0.26*
Agriculture	-0.07*	-0.03	-0.08	-0.14*	-0.09*	-0.1*
Others	-0.29*	-0.24*	-0.23*	-0.18*	-0.23*	-0.23*
Location						
Urban	-0.3*	-0.18*	-0.19*	-0.22*	-0.27*	-0.24*
Rural	-0.09*	-0.07*	-0.17*	-0.1*	-0.13*	-0.11*
ANC: nurse assisted	0.29*	0.24*	0.11*	0.33*	0.4*	0.39*
Age group						
15-24	0.26*	0.19*	0.11*	0.32*	0.4*	0.39*
25-49	0.3*	0.25*	0.11*	0.33*	0.39*	0.39*
Marital status						
Never	0.1	0.11*	0.09*	0.15	0.23*	0.16*
Currently / previously	0.29*	0.24*	0.11*	0.34*	0.4*	0.4*
Maternal occupation						
Not working	0.26*	0.2*	0.08*	0.46*	0.49*	0.47*
Professional / sales	0.16*	0.14*	0.04*	0.3*	0.35*	0.38*
Agriculture	0.21*	0.26*	0.12*	0.28*	0.26*	0.2*
Others	0.21*	0.14*	0.08*	0.29*	0.4*	0.38*
Location						
Urban	0.06*	0.06*	0.02	0.16*	0.17*	0.12*
Rural	0.24*	0.24*	0.12*	0.31*	0.41*	0.43*
ANC: government health facility	-0.02*	-0.03*	-0.04*	-0.04	-0.07*	-0.07*
Age group						
15-24	-0.01	-0.01	-0.02	-0.04	-0.05*	-0.06*
25-49	-0.02*	-0.04*	-0.05*	-0.04	-0.07*	-0.07*
Marital status						
Never	-0.08*	0.02	0	-0.15	-0.03	-0.05
Currently / previously	-0.02*	-0.03*	-0.04*	-0.04	-0.07*	-0.07*
Maternal occupation						
Not working	-0.01	-0.04*	-0.03*	-0.02	-0.08*	-0.08*
Professional / sales	-0.02	-0.02	-0.05*	-0.05*	-0.08*	-0.08*
Agriculture	0	-0.01	0	-0.06	0	-0.01
Others	-0.03	-0.05*	-0.02	-0.07	-0.1*	-0.09*
Location						
Urban	-0.03*	-0.01	-0.04*	-0.03	-0.1*	-0.07*
Rural	0	-0.02*	-0.02*	-0.03	-0.03*	-0.03*
ANC: private health facility	0.24*	0.3*	0.36*	0.2*	0.23*	0.27*
Age group						
15-24	0.15	0.05	0.21	0.23*	0.21*	0.27*
25-49	0.27*	0.34*	0.38*	0.19*	0.23*	0.25*
Marital status						
Never	0.44*	-0.1	0.26	0.37*	0.17	0.17*
Currently / previously	0.23*	0.33*	0.36*	0.19*	0.23*	0.27*
Maternal occupation						
Not working	0.12	0.42*	0.27*	0.24*	0.35*	0.37*
Professional / sales	0.17*	0.16	0.32*	0.18*	0.23*	0.27*
Agriculture	0.15	0.12	-0.08	0.2	0.01	0.01
Others	0.19*	0.32*	0.27*	0.24*	0.26*	0.33*
Location						
Urban	0.17*	0.11	0.26*	0.14*	0.21*	0.17*
Rural	0.09	0.32*	0.26*	0.18	0.15*	0.21*
ANC: 1st trimester	0.09*	0.09*	0.07*	-0.01	0.03	0.04
Age group						
15-24	0.08*	0.08	0.05	0.05	-0.03	0.01
25-49	0.09*	0.09*	0.07*	-0.03	0.04*	0.04
Marital status						
Never	0.03	0.01	0.08*	0.24	-0.06	0.02
Currently / previously	0.09*	0.1*	0.07*	-0.02	0.03	0.04
Maternal occupation						
Not working	0.13*	0.1*	0.08*	0.07	0.02	0.06
Professional / sales	0.07*	0.07*	0.05*	-0.05	0.06*	0.04
Agriculture	0.06*	0.06	0.02	0	0.01	-0.06
Others	0.04	0.04	0.05*	-0.03	0.1*	0.11*
Location						
Urban	0.06*	0.08*	0.07*	0.07	0.1*	0.09*
Rural	0.06*	0.08*	0.07*	-0.06	0.01	0.04*
ANC: +4 tetanus injection	0.05*	0.03*	0.02*	0.23*	0.27*	0.22*
Age group						
15-24	0.05*	0.04*	0.02*	0.24*	0.28*	0.22*
25-49	0.05*	0.03*	0.02*	0.23*	0.25*	0.22*
Marital status						
Never	0.01	0.07*	0.02	0.08	0.1*	0.07*
Currently / previously	0.05*	0.03*	0.02*	0.24*	0.27*	0.23*
Maternal occupation						
Not working	0.04	0.06*	0.01	0.33*	0.35*	0.29*
Professional / sales	0.02*	0.02*	0.01	0.24*	0.23*	0.21*
Agriculture	0.05*	0.02	0.01	0.14*	0.18*	0.1*
Others	0.03*	0.03*	0.01	0.21*	0.27*	0.19*
Location						
Urban	0.01	0.02*	0.01	0.11*	0.09*	0.06*
Rural	0.03*	0.03*	0.02*	0.2*	0.28*	0.23*
ANC: home	-0.26*	-0.25*	-0.1	-0.23*	-0.15*	0.05
Age group						
15-24	-0.07	-0.23	0.15	-0.23	-0.08	0.21*
25-49	-0.3*	-0.26	-0.19	-0.24*	-0.17*	0.01
Marital status^+^						
Never	0.08	-	-0.34	-0.31	-0.08	-0.02
Currently / previously	-0.27*	-	-0.07	-0.22*	-0.16*	0.06
Maternal occupation^+^						
Not working	-0.22	-	0.21	-0.33	-0.22*	0.05
Professional / sales	-0.27	-	-0.13	-0.22	-0.14*	0.09
Agriculture	-0.24	-	-0.06	0.05	0.01	0.11
Others	-0.43	-	-0.53	-0.24	-0.07	0.13
Location^+^						
Urban	-0.41	-	-0.18	-0.29*	-0.13	-0.02
Rural	-0.1	-	0.03	-0.12	-0.09*	0.11

Note: Magnitudes of regional variation is available in Additional file 1.

*p ≤ 0.01

^+^In 2008 the mean value of outcome is undefined.

Table 4 quantifies the degree of household wealth-related inequality in the use of delivery care services. Values of the concentration indices of home delivery declined in Ghana, increased in Nigeria, but remained negative in both countries. Closer observation reveals that the magnitude of inequality associated with the use of home delivery in Ghana was significant between regions in years 2003 and 2014. Significant differences in the six regions of Nigeria were also observed, and point estimates show that that the magnitude of inequality is not the same across geographical regions. In addition, results from Ghana data show the concentration of home delivery among poor households in rural and urban residences over the years. After 2008, there was no significant urban-rural differential in the magnitude of inequality in Ghana. However, in Nigeria, urban-rural inequality persisted. Though of equivalent magnitude, -0.09, the tests of the null hypothesis of equality indicate a significant difference in between women in rural and urban areas of Nigeria through the years. In 2008, the CI magnitude in Caesarean section increased in Nigeria from +0.49 to +0.58 but a decline in the CI magnitude was noted in Ghana from +0.45 to +0.30 for the same period. Significant differences between age groups were noted in Ghana and Nigeria after 2008; women who are 25-49 years consistently have greater CI magnitude in Caesarean section.

**Table 4:** Concentration Indices with Covariates: Ghana (years 2003, 2008 & 2014) and Nigeria (years 2003, 2008 & 2013)

Service type / covariate	Ghana			Nigeria		
	2003	2008	2014	2003	2008	2013
Delivery: home	-0.14*	-0.13*	-0.1*	-0.12*	-0.15*	-0.14*
Age group						
15-24	-0.13*	-0.09*	-0.07*	-0.09*	-0.12*	-0.11*
25-49	-0.15*	-0.14*	-0.1*	-0.12*	-0.16*	-0.15*
Marital status						
Never	-0.1*	-0.1*	-0.07*	-0.09*	-0.09*	-0.09*
Currently / previously	-0.14*	-0.13*	-0.1*	-0.11*	-0.15*	-0.14*
Maternal occupation						
Not working	-0.14*	-0.11*	-0.07*	-0.11*	-0.14*	-0.13*
Professional / sales	-0.12*	-0.09*	-0.06*	-0.13*	-0.17*	-0.16*
Agriculture	-0.06*	-0.09*	-0.06*	-0.08*	-0.08*	-0.06*
Others	-0.13*	-0.1*	-0.08*	-0.11*	-0.17*	-0.15*
Location						
Urban	-0.07*	-0.05*	-0.04*	-0.09*	-0.12*	-0.09*
Rural	-0.07*	-0.1*	-0.07*	-0.08*	-0.11*	-0.09*
Delivery: government health facility	0.10*	0.10*	0.06*	0.05*	0.07*	0.08*
Age group						
15-24	0.08*	0.07*	0.06*	0.04*	0.06*	0.07*
25-49	0.11*	0.11*	0.07*	0.06*	0.08*	0.08*
Marital status						
Never	0.04	0.1*	0.06*	0.01	0.03	0.03
Currently / previously	0.11*	0.1*	0.07*	0.05*	0.07*	0.08*
Maternal occupation						
Not working	0.08*	0.06*	0.04*	0.06*	0.07*	0.08*
Professional / sales	0.09*	0.07*	0.03*	0.05*	0.08*	0.08*
Agriculture	0.04*	0.07*	0.06*	0.03	0.05*	0.04*
Others	0.11*	0.07*	0.06*	0.05*	0.07*	0.07*
Location						
Urban	0.04*	0.03*	0.01	0.03	0.03*	0.03*
Rural	0.05*	0.08*	0.06*	0.03*	0.06*	0.06*
Delivery: private health facility	0.04*	0.03*	0.03*	0.06*	0.08*	0.07*
Age group						
15-24	0.04*	0.02	0.01	0.05*	0.05*	0.05*
25-49	0.04*	0.03*	0.03*	0.07*	0.09*	0.07*
Marital status						
Never	0.06	0	0.01	0.09*	0.06*	0.05*
Currently / previously	0.04*	0.03*	0.03*	0.06*	0.08*	0.07*
Maternal occupation						
Not working	0.06*	0.06*	0.03	0.06*	0.06*	0.05*
Professional / sales	0.03*	0.02	0.04*	0.07*	0.09*	0.08*
Agriculture	0.01*	0.02*	0	0.06*	0.03*	0.01
Others	0.02*	0.02	0.02	0.06*	0.09*	0.07*
Location						
Urban	0.03	0.01	0.03*	0.06*	0.09*	0.06*
Rural	0.01*	0.02*	0.01	0.04*	0.04*	0.03*
Birth assistance: doctor	0.03*	0.04*	0.05*	0.04*	0.06*	0.05*
Age group						
15-24	0.03*	0.01	0.02	0.03*	0.03*	0.03*
25-49	0.04*	0.05*	0.06*	0.04*	0.06*	0.06*
Marital status						
Never	0.06	0.03	0.04*	0.11*	0.03*	0.03*
Currently / previously	0.03*	0.04*	0.05*	0.03*	0.06*	0.05*
Maternal occupation						
Not working	0.05*	0.06*	0.03*	0.03*	0.05*	0.04*
Professional / sales	0.04*	0.04*	0.06*	0.05*	0.07*	0.06*
Agriculture	0.01*	0.01	0.01	0.04	0.01*	0.02*
Others	0.04*	0.04*	0.06*	0.03*	0.06*	0.05*
Location						
Urban	0.04*	0.04*	0.05*	0.05*	0.08*	0.07*
Rural	0.01	0.02*	0.03*	0.01*	0.02*	0.02*
C Section	0.45*	0.30*	0.31*	0.49*	0.58*	0.49*
Age group						
15-24	0.37	0.05	0.07	0.22	0.46*	0.33*
25-49	0.47*	0.34*	0.32*	0.54*	0.59*	0.52*
Marital status						
Never	0.38	0.26	0.2	0.53	0.57*	0.27
Currently / previously	0.45*	0.31*	0.32*	0.48*	0.58*	0.5*
Maternal occupation						
Not working	0.36	0.26	0.15	0.5*	0.6*	0.59*
Professional / sales	0.45*	0.19*	0.28*	0.44*	0.55*	0.49*
Agriculture	0.14	0.28	0.11	0.61	0.19	0.28
Others	0.43	0.25	0.27*	0.45	0.46*	0.43*
Location						
Urban	0.36*	0.11	0.19*	0.36*	0.41*	0.36*
Rural	0.14	0.38*	0.3*	0.29	0.52*	0.37*

Note: Magnitudes of regional variation is available in Additional file 1.

*p ≤ 0.01

## Discussion

The concentration curve and associated indices estimated in this study permit the investigation of the progress made in reducing inequalities in access to reproductive health care services among women in Ghana and Nigeria. These indicators of use of family planning services, antenatal and delivery care services show that the use of some services is inequitably distributed and that there are differences in the size of inequality within socioeconomic groups of women. Specifically, the use of antenatal care at government facilities, health worker’s assistance during pregnancy outside a facility, childbirth at home is distributed unequally and advantaging women in poor households, while the use of family planning information via TV or print media, antenatal care at private facilities are advantaging women in wealthier households in both Ghana and Nigeria. In Nigeria alone, women in richer households have considerably more unmet needs for family planning.

The variation in the magnitudes of inequality across socioeconomic groups within and between the countries (measured by the concentration indices), is also necessary to understand how the determinants of inequalities differ. We find that nearly all indicators of use of reproductive health care services in Ghana indicated a shift towards the equity line, indicating a decline in inequality. However, equity improvement was not observed in doctor-assisted births, antenatal care provided at non-facility formations, government and private facilities. In Nigeria, indicators examined showed mixed shifts, with mostly non-pro-poor changes over the years observed. This was specifically the case for antenatal care at private facilities, antenatal care in government facilities, non-facility formations for antenatal care, family planning information via TV or print media, unmet needs for family planning, and childbirth at home. The most substantial change in the magnitude of inequality, a decrease, was noted for the use of modern contraceptives during years 2003 – 2008 in both Ghana and Nigeria. The most substantial change in antenatal care was observed in 2003 – 2008 for the indicators antenatal care at government health facilities in Ghana and antenatal care in the 1st trimester in Nigeria. Among indicators of delivery care, the most substantial change in the magnitude of inequality in home delivery in Ghana occurred among women in poor households in the period 2008 – 2014. In Nigeria, a change in the magnitude of inequality was most evident in delivery at a government health facility and private health facility during the period 2003-2008.

### Family planning services

We find that use of family planning information via TV or print media is unequally distributed in favor of women in wealthy households of Ghana, specifically, those with agricultural livelihood and women who live in Upper East region. This is unsurprising since wealth is correlated with education which facilitates access and assimilation of information [46]. This finding supports previous research that showed that higher socioeconomic status improves the use of family planning media messages [47]. Although studies show that few people get family planning information via media messages [48]. Another study found that access to family planning information via television or print medium is in disfavor of women in lower socioeconomic strata [49]. However, access to family planning information via media messages about reproductive health care services has mixed results in promoting access in Africa [23, 49, 50]. We also find that unwanted births, indicated by unmet needs for family planning, are concentrated in wealthy households of Nigeria and occur most among women who are not working or living in rural residences. This finding suggests that there is a high need for contraception among women in rural economically advantaged households. Studies show that wage-earning or economically self-sufficient women are more likely to seek contraception, though modern means of preventing unwanted births could be inaccessible in cultural and religious societies [48, 51]. Finally, regarding access to family planning services, while economic status does preclude women from making sole reproductive decisions it could, however, initiate a demand for contraception [52–54].

### Antenatal care services

We find that, in both Ghana and Nigeria, women in poor households have increasingly become disadvantaged as inequality in use of antenatal care at private facilities increased in favor of their counterparts in wealthy households. A study carried out in Ghana using DHS data from years 1988 – 2008 found a similar increase. However, this study did not disaggregate antenatal care by type of provider [36]. It is reported that wealthier women are better able to overcome barriers of informal payments of cash or kind and are less likely to encounter negative health workers attitudes often seen in private health care facilities [55, 56]. Other studies have shown that wealth-related inequalities in the use of antenatal care have increased in the past years [36, 57]. Results from our study further suggest that the use of health worker’s assistance during pregnancy outside a facility in both countries became less equitable; women in poor households in urban areas or with professional/sales occupation use such assistance more frequently. Professional occupation and urban area residents are generally thought to have better access to good quality reproductive health care services, given their knowledge and the service availability accessible to these women to draw from [58–61]. Our finding deviates from other studies which suggest that women in these groups access better antenatal care services. To explain the variation in the use of health worker’s assistance during pregnancy outside a facility, studies have also shown that transport and health facilities waiting time may facilitate the use of such assistance or deter the use of modern antenatal care services among women in these categories [60, 62–65]. Specifically, the observed change in home antenatal care from prominence among poor to richer households in Nigeria is unexpected. AF Fagbamigbe and ES Idemudia [66] also noted non-use of antenatal care among the wealthier women during pregnancy and suggest that not only poverty but also other factors like personality and view on the quality of services are relevant. It is also plausible that these are a response to increased pressure on resources in government or other maternal care formations [64]. Unfortunately, there is no information on the quality of care in the DHS data.

The finding that antenatal care in government facilities in both countries is pro-poor and consistently changing to the advantage of women in poor households was observed in different years. No effect was observed among women in the agrarian sector, however. One study report that the use of antenatal care improved among women in Ghana, and, though economic challenges are being surmounted, it may be delayed among women in agricultural occupations [36]. Other studies report pro-wealthy inequality changes in antenatal care use among women in Ghana and Nigeria between 2003 and 2008 [19, 57]. In addition, urban-rural inequality in the use of antenatal care at government facilities was observed in the later years in Nigeria but diminished in Ghana. In Nigeria, we find no evidence that rural women in poor households seek antenatal health care services at government facilities. Other studies found unequal use of antenatal care services to the detriment of women in rural households [62, 66]. A study of Nigeria’s midwives service scheme found insignificant success in rural areas attributable to pro-wealthy resource distribution [24]. Nonetheless, the observed diminished urban-rural differential to benefit women in poor households in Ghana has been partially credited to improvements in infrastructure and maternal health care services [36].

### Delivery care services

Our study finds that childbirth at home persists among women in poor households although overall inequality magnitude appears to have declined in Ghana while it has increased in Nigeria, there are substantial geographical variations. It appears that by 2014 inequality became notable among women in all seven regions of Ghana. In a 2005 research on the free delivery care policy in Central and Volta regions of Ghana, an increase in facility delivery and a decline in home delivery was reported [26]. Another study found that coverage of the doorstep community-based health planning and services program in Ghana was substantial in mainly the Upper East region [23]. A separate study carried out among Nigerian women in 2004 did not find a substantial increase in institutional delivery facilities in Nigeria despite the midwives service scheme [24]. Evidence of substantial pro-rich inequality between the Northern and Southern regions was observed in Nigeria, while delivery at government health facilities favored women in the Northern regions. Delivery at a private health facility is more inequitable among the richer households in the Southern regions. We find persisting rural-urban disparities associated with childbirth at a government health facility in Nigeria, but not in the later years in Ghana, that is 2008 and 2014. Other research did not find evidence of rural-urban differences in the shift from home to health facilities in Ghana and Nigeria [67]. The observed inequalities among women who have childbirth at home suggest that implemented health policies such as the community-based health planning and services initiative in Ghana, a free delivery scheme in Ghana, the midwives service scheme in Nigeria and insurance schemes in both counties have not substantially reduced inequality in home birth among women. This is also confirmed in other studies [16, 36, 57].

The finding that Caesarean section in both Ghana and Nigeria is pro-wealthy is not surprising. Our findings are in line with the findings of previous studies that Caesarean section remains under-provided for women in poor households in both countries [16]. The trend suggests that coverage gap in both countries remains relatively high. In addition, the equity trend observed in Nigeria suggests top inequity, indicating that the increase in the CI magnitude related to Caesarean section is extremely high.

### Study limitation

The cross-sectional design of the study implies that we can show associations without concluding about causal relationships. Other methodological limitations of the study include recall bias since the survey collects events over a five-year period. A limitation regarding the country comparisons concerns the fact that inequalities were investigated based on the position of women in the distribution of household wealth in their own country. We recognize that a woman who is poor by Ghana standards may be better off in Nigeria. Also, we recognize that measures of coverage gap [68], which we have not analyzed, are as equally important as the equity gap evidenced in this paper. Coverage gap refers to the difference between the targeted and actual use of essential health care services by the population, while equity gap indicates the distribution of the services use across the wealth-based population groups [68]. Thus, the underutilization of reproductive care is not directly addressed in our equity study. However, the study has some strengths as well. In particular, the merging of important indicators of maternal care improved the ability to capture the different categories of reproductive care. In addition, we use a generalized concentration index as a measure of inequality, which is not sensitive to outcome measures because it quantifies the absolute differences in health between income groups. Finally, the measurement of the magnitudes of inequalities over different years gives indications about the changing horizontal inequalities which are country and time specific.

## Conclusion

Inequality in the use of family planning, antenatal and delivery care services among women of reproductive health care services in both Ghana and Nigeria have persisted over the years despite efforts and have provided little improvement for women in poor households. The results show that inequality increased in case of antenatal care at private facilities, health worker’s assistance during pregnancy outside a facility, antenatal care in government facilities, home births, aspects of reproductive health care services in both Ghana and Nigeria, and unmet need for family planning in Nigeria. Changes in inequality were mostly to the disadvantage of women in poorer households in Nigeria but less in Ghana. The changes in inequality had little effect on improving the use of quality reproductive health care services among women particularly those in poor households. Furthermore, the disambiguation of indicators of the use of reproductive health services shows the extent of the progress made in eliminating unequal access among sociodemographic groups. Also, disaggregation of determinants of access indicated notable horizontal inequalities among women of different socioeconomic groups in Ghana and Nigeria.

The gains made in reducing inequality access to reproductive health care services have eroded over time. This implies that the sustainability of health initiatives to reduce inequalities needs to be addressed. Ghana’s health initiatives need to take a pro-poor concept and Nigeria’s an accelerated implementation across the population to bring about the decline in inequality in access.

## Additional files

Additional file 1: Examining the trend of inequality. A1 - Definition of indicators used in the analysis, Ghana. Grouping description of outcome variable. A2 - Definition of indicators used in the analysis, Nigeria. Grouping description of outcome variable. A3 - Definition of independent variables used in the analysis. Coding of the dependent and control variables. A4 - Socio-demographic characteristics: Ghana (years 2003, 2008 & 2014) and Nigeria (years 2003, 2008 & 2013). Distribution of the independent variables. B - Supplementary data: Distribution of outcome variables (poorest 20%, richest 20% and total number of women). Distribution of the control variables. C - Concentration Indices with Covariates: Ghana (years 2003, 2008 & 2014) and Nigeria (years 2003, 2008 & 2013). Concentration Indices with Covariates and F-test result. D – Concentration Indices with Covariates: Ghana (years 2003, 2008 & 2014) and Nigeria (years 2003, 2008 & 2013). Concentration Indices with Covariates and F-test result. E – Concentration Indices with Covariates: Ghana (years 2003, 2008 & 2014) and Nigeria (years 2003, 2008 & 2013). Concentration Indices with Covariates and F-test result. (PDF 1384 kb)

Additional file 2: **Figure S1.** Concentration curves of use of family planning Ghana (Years 2003, 2008, 2014). (PDF 26 kb)

Additional file 3: **Figure S2.** Concentration curves of use of family planning Nigeria (Years 2003, 2008, 2013). (PDF 26 kb)

Additional file 4: **Figure S3.** Concentration curves of use of Antenatal care, Ghana (Years 2003, 2008, 2013). (PDF 924 kb)

Additional file 5: **Figure S4.** Concentration curves of use of Antenatal care, Nigeria (Years 2003, 2008, 2014). (PDF 1208 kb)

Additional file 6: **Figure S5.** Concentration curves of use of Delivery care, Ghana (Years 2003, 2008, 2014). (PDF 899 kb)

Additional file 7: **Figure S6.** Concentration curves of use of delivery care, Nigeria (Years 2003, 2008, 2013). (PDF 393 kb)
