# Supplementary material for: Examining trends in inequality in the use of reproductive health care services in Ghana and Nigeria
Source: BMC Pregnancy Childbirth. 2018 Dec 13;18:492. doi: 10.1186/s12884-018-2102-9 (PMC6293518; doi:10.1186/s12884-018-2102-9)
Supplement: Supplementary file 1 — Examining the trend of inequality. A1 - Definition of indicators used in the analysis, Ghana. Grouping description of outcome variable. A2 - Definition of indicators used in the analysis, Nigeria. Grouping description of outcome variable. A3 - Definition of independent variables used in the analysis. Coding of the dependent and control variables. A4 - Socio-demographic characteristics: Ghana (years 2003, 2008 & 2014) and Nigeria (years 2003, 2008 & 2013). Distribution of the independent variables. B - Supplementary data: Distribution of outcome variables (poorest 20%, richest 20% and total number of women). Distribution of the control variables. C - Concentration Indices with Covariates: Ghana (years 2003, 2008 & 2014) and Nigeria (years 2003, 2008 & 2013). Concentration Indices with Covariates and F-test result. D – Concentration Indices with Covariates: Ghana (years 2003, 2008 & 2014) and Nigeria (years 2003, 2008 & 2013). Concentration Indices with Covariates and F-test result. E – Concentration Indices with Covariates: Ghana (years 2003, 2008 & 2014) and Nigeria (years 2003, 2008 & 2013). Concentration Indices with Covariates and F-test result. (PDF 1384 kb) [file 12884_2018_2102_MOESM1_ESM.pdf]

Title of data: A1 - Definition of indicators used in the analysis, Ghana
Description of data: Grouping description of outcome variable

Outcome	GH 2003	GH 2008	GH 2014
**Family planning**			
Family planning info: Health facility	At health facility, told of family planning	At health facility, told of family planning	At health facility, told of family planning
Family planning worker visit	Visited by FP worker last 12m	Visited by FP worker last 12m	Visited by FP worker last 12m
Family planning: TV	Heard FP on TV last months	Heard FP on TV last months	Heard FP on TV last months
Family planning: Print	Heard FP newspaper last months	Heard FP newspaper last months	Heard FP newspaper last months
Modern contraceptive	Current use by method type	Current use by method type	Current use by method type
Information on pregnancy complication	Told about pregnancy complications	Told about pregnancy complications	Told about pregnancy complications
Family planning: unmet need	Wanted last child	Wanted last child	Wanted last child
**Antenatal care**			
ANC: nurse assisted	Assistance: nurse/midwife	Assistance: nurse/midwife	Assistance: nurse/midwife
ANC: government health facility	Antenatal care: govt. hospital<br>Antenatal care: govt. health center<br>Antenatal care: govt. health post<br>Antenatal care: public mobile clinic<br>Antenatal care: other public	Antenatal care: govt. hospital<br>Antenatal care: govt. health center<br>Antenatal care: govt. health post<br>Antenatal care: public mobile clinic<br>Antenatal care: other public	Antenatal care: govt. hospital<br>Antenatal care: govt. health center<br>Antenatal care: govt. health post<br>Antenatal care: public mobile clinic<br>Antenatal care: other public
ANC: Private health facility	Antenatal care: pvt. hospital/clinic<br>Antenatal care: pvt. mobile clinic<br>Antenatal care: Maternity home<br>Antenatal care: other private	Antenatal care: pvt. hospital/clinic<br>Antenatal care: pvt. mobile clinic<br>Antenatal care: Maternity home<br>Antenatal care: other private	Antenatal care: pvt. hospital/clinic<br>Antenatal care: FP/PPAG clinic<br>Antenatal care: mobile clinic<br>Antenatal care: other private
ANC: 1st trimester	Timing of 1st antenatal check	Timing of 1st antenatal check	Timing of 1st antenatal check
ANC: 4+ tetanus injection	Tetanus injections bef. birth	Tetanus injections bef. birth	Tetanus injections bef. birth
ANC: Home	Antenatal care: your home<br>Antenatal care: other home<br>Antenatal care: TBA's home	Antenatal care: respondent's home<br>Antenatal care: other home<br>Antenatal care: other	Antenatal care: respondent's home<br>Antenatal care: other home
Health worker's assistance during pregnancy outside a facility	Assistance: auxiliary midwife<br>Assistance: trad.birth attend.<br>Assistance: relative, friend<br>Assistance: no one<br>Assistance: other resp (uncod)	Assistance: auxiliary midwife<br>Assistance: community health officer/nurse<br>Assistance: traditional birth attendant (trained)<br>Assistance: community/village health volunteer<br>Assistance: traditional practitioner<br>Assistance: other<br>Assistance: no one	Assistance: auxiliary midwife<br>Assistance: community/village health volunteer<br>Assistance: traditional practitioner<br>Assistance: other<br>Assistance: no one
**Delivery care**			
Delivery: home	Place of delivery: Respondents home<br>Other home<br>TBA's home	Place of delivery: Respondents home<br>Other home<br>TBA's home	Place of delivery: Respondents home<br>Other home<br>TBA's home
Delivery: government health facility	Place of delivery: Govt. hospital<br>Govt. health center<br>Govt. health post	Place of delivery: Govt. hospital<br>Govt. health center<br>Govt. health post	Place of delivery: Govt. hospital<br>Govt. health center<br>Govt. health post
Delivery: private health facility	Place of delivery: Private hosp/clinic<br>Maternity home<br>OTHER	Place of delivery: Private hosp/clinic<br>Maternity home<br>OTHER	Place of delivery: Private hosp/clinic<br>Maternity home<br>OTHER
Birth assistance: Doctor	Assistance: doctor	Assistance: doctor	Assistance: doctor

Title of data: A2 - Definition of indicators used in the analysis, Nigeria
Description of data: Grouping description of outcome variable

Outcome	NG 2003	NG 2008	NG 2013
**Family planning**	**Family planning**		
Family planning info: Health facility	At health facility, told of family planning	At health facility, told of family planning	At health facility, told of family planning
Family planning worker visit	Visited by FP worker last 12m	Visited by FP worker last 12m	Visited by FP worker last 12m
Family planning: TV	Heard FP on TV last months	Heard FP on TV last months	Heard FP on TV last months
Family planning: Print	Heard FP newspaper last months	Heard FP newspaper last months	Heard FP newspaper last months
Modern contraceptive	Current use by method type	Current use by method type	Current use by method type
Information on pregnancy complication	Told about pregnancy complications	Told about pregnancy complications	Told about pregnancy complications
Family planning: unmet need	Wanted last child	Wanted last child	Wanted last child
**Antenatal care**			
ANC: nurse assisted	Assistance: nurse/midwife	Assistance: nurse/midwife	Assistance: nurse/midwife
ANC: government health facility	Antenatal care: govt. hospital<br>Antenatal care: govt. health center<br>Antenatal care: govt. health post<br>Antenatal care: public mobile clinic<br>Antenatal care: other public	Antenatal care: govt. hospital<br>Antenatal care: govt. health center<br>Antenatal care: govt. health post/ dispensary<br>Antenatal care: other public	Antenatal care: government hospital<br>Antenatal care: government health center<br>Antenatal care: government health post/ dispensary<br>Antenatal care: other public sector
ANC: Private health facility	Antenatal care: pvt. hospital/clinic<br>Antenatal care: other private	Antenatal care: pvt. hospital/clinic<br>Antenatal care: other private	Antenatal care: private hospital/clinic<br>Antenatal care: other private medical sector
ANC: 1st trimester	Timing of 1st antenatal check	Timing of 1st antenatal check	Timing of 1st antenatal check
ANC: 4+ tetanus injection	Tetanus injections bef. birth	Tetanus injections bef. birth	Tetanus injections bef. birth
ANC: Home	Antenatal care: your home<br>Antenatal care: other home<br>Antenatal care: other	Antenatal care: your home<br>Antenatal care: other home<br>Antenatal care: other	Antenatal care: respondent's home<br>Antenatal care: other home<br>Antenatal care: other
Health worker's assistance during pregnancy outside a facility	Assistance: auxiliary midwife<br>Assistance: trad.birth attend.<br>Assistance: relative, friend<br>Assistance: other resp (uncod)<br>Assistance: no one	Assistance: auxiliary midwife<br>Assistance: trad.birth attend.<br>Assistance: relative, friend Assistance: other resp (uncod)<br>Assistance: no one.	Assistance: auxiliary midwife<br>Assistance: community extension health worker<br>Assistance: traditional birth attendant<br>Assistance: relative/ friend<br>Assistance: other Assistance: no one
**Delivery care**			
Delivery: home	Place of delivery: Respondents home<br>Other home<br>TBA's home	Place of delivery: Respondents home<br>Other home<br>TBA's home	Place of delivery: Respondents home<br>Other home<br>TBA's home
Delivery: government health facility	Place of delivery: Govt. hospital<br>Govt. health center<br>Govt. health post	Place of delivery: Govt. hospital<br>Govt. health center<br>Govt. health post	Place of delivery: Govt. hospital<br>Govt. health center<br>Govt. health post
Delivery: private health facility	Place of delivery: Private hosp/clinic<br>Maternity home<br>OTHER	Place of delivery: Private hosp/clinic<br>Maternity home<br>OTHER	Place of delivery: Private hosp/clinic<br>Maternity home<br>OTHER
Birth assistance: Doctor	Assistance: doctor	Assistance: doctor	Assistance: doctor

Title of data: A3 - Definition of independent variables used in the analysis
Description of data: Coding of the dependent and control variables

Independent variable	Questions asked	Coding
**Age group**	**Age 5-year groups**	Categorical
	15-19	
15-24	20-24	
	25-29	
	30-34	
	35-39	
	40-44	
25-49	45-49	
**Marital status**	**Current marital status**	Categorical
never	Never married	
	Married	
	Living together	
	Widowed	
	Divorced	
now / then	Not living together	
**Maternal occupation**	**Respondent's occupation**	Categorical
not working	Not working	
	Professional/technical/managerial	
	Clerical	
Professional / sales	Sales	
	Agricultural - self employed	
Agriculture	Agricultural - employee	
	Household and domestic	
	Services	
	Skilled manual	
Others	Unskilled manual	
**Location**	**Type of place of residence**	Categorical
urban		
rural		
Region (Ghana)	Region	Categorical
Region (Nigeria)	Region	Categorical

Title of data: A4 - Socio-demographic characteristics: Ghana (years 2000, 2005 & 2014) and Nigeria (years 2003, 2008 & 2013)
Description of data: Distribution of the independent variables

	GHANA			NIGERIA		
**Maternal characteristic**	**2003**	**2008**	**2014**	**2003**	**2008**	**2013**
Age group						
15-24	649 (23.4)	518 (24.1)	923 (21.5)	1090 (28.9)	4779 (26.5)	5180 (25.7)
25-49	2128 (76.6)	1629 (75.9)	3371 (78.5)	2685 (71.1)	13249 (73.5)	15012 (74.3)
Marital status						
Never	87 (3.1)	117 (5.4)	363 (8.5)	103 (2.7)	455 (2.5)	538 (2.7)
Now / then	2690 (96.9)	2030 (94.6)	3931 (91.5)	3672 (97.3)	17572 (97.5)	19654 (97.3)
Maternal occupation						
Not working	292 (10.6)	219 (10.3)	759 (17.7)	1286 (34.1)	5704 (31.8)	5877 (29.3)
Professional / Sales	689 (25.0)	753 (35.3)	1603 (37.4)	1556 (41.2)	6314 (35.2)	8498 (42.3)
Agriculture	1260 (45.7)	812 (38.0)	1316 (30.7)	584 (15.5)	3508 (19.6)	2458 (12.2)
Others	519 (18.8)	351 (16.4)	608 (14.2)	349 (9.2)	2404 (13.4)	3240 (16.1)
Location						
Urban	817 (29.4)	763 (35.5)	1778 (41.4)	1350 (35.8)	4825 (26.8)	6790 (33.6)
Rural	1960 (70.6)	1384 (64.5)	2516 (58.6)	2425 (64.2)	13203 (73.2)	13402 (66.4)
Region (Ghana)						
Western	237 (8.5)	189 (8.8)	431 (10.0)	-	-	-
Central	168 (6.0)	158 (7.4)	436 (10.2)	-	-	-
Greater Accra	264 (9.5)	210 (9.8)	354 (8.2)	-	-	-
Volta	202 (7.3)	181 (8.4)	346 (8.1)	-	-	-
Eastern	228 (8.2)	187 (8.7)	397 (9.2)	-	-	-
Ashanti	423 (15.2)	318 (14.8)	420 (9.8)	-	-	-
Brong Ahafo	337 (12.1)	207 (9.6)	490 (11.4)	-	-	-
Northern	429 (15.4)	306 (14.3)	622 (14.5)	-	-	-
Upper West	265 (9.5)	181 (8.4)	434 (10.1)	-	-	-
Upper East	224 (8.1)	210 (9.8)	364 (8.5)	-	-	-
Region (Nigeria)						
North Central	-	-	-	645 (17.1)	3350 (18.6)	3095 (15.3)
North East	-	-	-	867 (23.0)	3972 (22.0)	4001 (19.8)
North West	-	-	-	1125 (29.8)	4888 (27.1)	6206 (30.7)
South East	-	-	-	329 (8.7)	1454 (8.1)	1724 (8.5)
South South	-	-	-	380 (10.1)	2101 (11.7)	2500 (12.4)
South West	-	-	-	429 (11.4)	2263 (12.6)	2666 (13.2)
Total	2777	2147	4294	3775	18028	20192

Title of data: B - Supplementary data: Distribution of outcome variables (poorest 20%, richest 20% and total number of women)
Description of data: Distribution of the control variables

**Ghana**		**2003**					**2008**					**2014**				
		Poorest	%	Richest	%	All women	Poorest	%	Richest	%	All women	Poorest	%	Richest	%	All women
Family planning info: Health facility	No	141	18.98	178	23.96	743	115	15.13	190	25	760	312	22.29	246	17.57	1400
	Yes	147	18.8	183	23.4	782	116	20.03	123	21.24	579	354	24.2	220	15.04	1463
	Total	288	18.89	361	23.67	1525	231	17.25	313	23.38	1339	666	23.26	466	16.28	2863
Family planning worker visit	No	492	20.95	482	20.52	2349	350	19.73	363	20.46	1774	854	23.47	586	16.1	3639
	Yes	80	20.36	63	16.03	393	73	20.8	68	19.37	351	174	26.56	76	11.6	655
	Total	572	20.86	545	19.88	2742	423	19.91	431	20.28	2125	1028	23.94	662	15.42	4294
FP Info: TV	No	507	29.02	98	5.61	1747	401	28.64	90	6.43	1400	889	35.24	141	5.59	2523
	Yes	67	6.71	447	44.79	998	23	3.17	341	46.97	726	139	7.85	521	29.42	1771
	Total	574	20.91	545	19.85	2745	424	19.94	431	20.27	2126	1028	23.94	662	15.42	4294
FP Info: Print	No	567	23.29	350	14.38	2434	420	21.19	350	17.66	1982	1018	24.7	581	14.1	4121
	Yes	6	1.95	195	63.31	308	4	2.78	81	56.25	144	10	5.78	81	46.82	173
	Total	573	20.9	545	19.88	2742	424	19.94	431	20.27	2126	1028	23.94	662	15.42	4294
Modern contraceptive	No	23	13.07	52	29.55	176	5	4.2	43	36.13	119	22	15.17	50	34.48	145
	Yes	66	12.87	158	30.8	513	58	15.59	98	26.34	372	220	19.98	152	13.81	1101
	Total	89	12.92	210	30.48	689	63	12.83	141	28.72	491	242	19.42	202	16.21	1246
Information on pregnancy complication	No	251	24.25	152	14.69	1035	177	27.87	93	14.65	635	181	24.97	72	9.93	725
	Yes	239	16.07	385	25.89	1487	213	15.24	331	23.68	1398	791	23.11	585	17.09	3423
	Total	490	19.43	537	21.29	2522	390	19.18	424	20.86	2033	972	23.43	657	15.84	4148

Ghana		2003					2008					2014				
		Poorest	%	Richest	%	All women	Poorest	%	Richest	%	All women	Poorest	%	Richest	%	All women
Family planning: unmet need	No	374	22.89	337	20.62	1634	290	21.9	287	21.68	1324	769	25.43	500	16.53	3024
	Yes	197	17.91	207	18.82	1100	133	16.58	144	17.96	802	259	20.39	162	12.76	1270
	Total	571	20.89	544	19.9	2734	423	19.9	431	20.27	2126	1028	23.94	662	15.42	4294
Health worker's assistance during pregnancy outside a facility	No	110	9.81	445	39.7	1121	232	16.84	365	26.49	1378	783	21.82	624	17.39	3588
	Yes	461	28.6	99	6.14	1612	190	25.47	66	8.85	746	245	34.7	38	5.38	706
	Total	571	20.89	544	19.9	2733	422	19.87	431	20.29	2124	1028	23.94	662	15.42	4294
ANC: nurse assisted	No	466	28.59	125	7.67	1630	338	31.35	86	7.98	1078	541	34.31	124	7.86	1577
	Yes	105	9.52	419	37.99	1103	84	8.03	345	32.98	1046	487	17.92	538	19.8	2717
	Total	571	20.89	544	19.9	2733	422	19.87	431	20.29	2124	1028	23.94	662	15.42	4294
ANC: government health facility	No	45	15.46	87	29.9	291	19	8.37	81	35.68	227	34	9.42	133	36.84	361
	Yes	446	19.96	451	20.19	2234	375	20.73	341	18.85	1809	941	24.75	526	13.83	3802
	Total	491	19.45	538	21.31	2525	394	19.35	422	20.73	2036	975	23.42	659	15.83	4163
ANC: Private health facility	No	460	20.6	431	19.3	2233	382	20.6	345	18.61	1854	939	24.61	526	13.78	3816
	Yes	31	10.62	107	36.64	292	12	6.59	77	42.31	182	36	10.37	133	38.33	347
	Total	491	19.45	538	21.31	2525	394	19.35	422	20.73	2036	975	23.42	659	15.83	4163
ANC: 1st trimester	No	285	22.44	203	15.98	1270	208	24.21	126	14.67	859	381	26.53	160	11.14	1436
	Yes	204	16.36	334	26.78	1247	180	15.38	294	25.13	1170	593	21.76	499	18.31	2725
	Total	489	19.43	537	21.33	2517	388	19.12	420	20.7	2029	974	23.41	659	15.84	4161
ANC: +4 tetanus injection	No	145	33.56	30	6.94	432	83	32.17	18	6.98	258	160	32.52	42	8.54	492

Ghana		2003					2008					2014				
		Poorest	%	Richest	%	All women	Poorest	%	Richest	%	All women	Poorest	%	Richest	%	All women
	Yes	421	18.67	493	21.86	2255	335	18.19	407	22.1	1842	858	22.81	616	16.37	3762
	Total	566	21.06	523	19.46	2687	418	19.9	425	20.24	2100	1018	23.93	658	15.47	4254
ANC: Home	No	476	19.23	537	21.7	2475	384	19.26	419	21.01	1994	972	23.45	656	15.83	4145
	Yes	15	30	1	2	50	10	23.81	3	7.14	42	3	16.67	3	16.67	18
	Total	491	19.45	538	21.31	2525	394	19.35	422	20.73	2036	975	23.42	659	15.83	4163
Home delivery	No	121	10.06	466	38.74	1203	108	8.88	384	31.58	1216	560	18.02	631	20.3	3108
	Yes	453	29.38	79	5.12	1542	316	34.69	47	5.16	911	468	39.46	31	2.61	1186
	Total	574	20.91	545	19.85	2745	424	19.93	431	20.26	2127	1028	23.94	662	15.42	4294
Delivery: government health facility	No	470	26.18	184	10.25	1795	325	29.73	121	11.07	1093	495	33.4	146	9.85	1482
	Yes	104	10.95	361	38	950	99	9.57	310	29.98	1034	533	18.95	516	18.35	2812
	Total	574	20.91	545	19.85	2745	424	19.93	431	20.26	2127	1028	23.94	662	15.42	4294
Delivery: private health facility	No	560	22.37	441	17.62	2503	416	21.38	357	18.35	1946	1001	25.03	547	13.68	3999
	Yes	14	5.79	104	42.98	242	8	4.42	74	40.88	181	27	9.15	115	38.98	295
	Total	574	20.91	545	19.85	2745	424	19.93	431	20.26	2127	1028	23.94	662	15.42	4294
Birth assistance: Doctor	No	556	21.77	442	17.31	2554	412	21.54	330	17.25	1913	974	25.65	482	12.69	3797
	Yes	15	8.38	102	56.98	179	10	4.74	101	47.87	211	54	10.87	180	36.22	497
	Total	571	20.89	544	19.9	2733	422	19.87	431	20.29	2124	1028	23.94	662	15.42	4294
Caesarean section	No	561	21.32	487	18.51	2631	415	20.85	373	18.74	1990	972	25.54	503	13.22	3806
	Yes	10	10.00	56	56.00	100	9	6.67	57	42.22	135	56	11.48	159	32.58	488

**Ghana**		**2003**					**2008**					**2014**				
		Poorest	%	Richest	%	All women	Poorest	%	Richest	%	All women	Poorest	%	Richest	%	All women
	Total	571	20.91	543	19.88	2731	424	19.95	430	20.24	2125	1028	23.94	662	15.42	4294

**Nigeria**		**2003**					**2008**					**2013**				
		Poorest	%	Richest	%	All women	Poorest	%	Richest	%	All women	Poorest	%	Richest	%	All women
Family planning info: Health facility	No	153	19.64	297	29.66	1135	353	19.43	833	43.89	2726	545	19.63	707	5.224	3385
	Yes	51	19.64	176	29.54	469	108	18.25	810	41.57	1798	183	18.88	852	8.731	2743
	Total	204	19.64	473	29.42	1604	461	17.08	1643	39.25	4524	728	18.14	1559	12.24	6128
Family planning worker visit	No	707	19.64	659	29.3	3476	3489	15.9	3115	36.93	16677	4802	17.39	2338	15.75	17612
	Yes	20	19.64	84	37.84	222	67	6.5	444	43.11	1030	150	6.06	739	29.83	2477
	Total	727	19.64	743	20.09	3698	3556	20.08	3559	20.1	17707	4952	24.65	3077	15.32	20089
FP Info: TV	No	703	19.64	370	12.29	3011	3534	23.99	1689	11.47	14731	4909	29.41	1570	9.41	16691
	Yes	23	19.64	373	54.37	686	39	1.28	1871	61.24	3055	69	1.99	1511	43.49	3474
	Total	726	19.64	743	20.1	3697	3573	20.09	3560	20.02	17786	4978	24.69	3081	15.28	20165
FP Info: Print	No	709	21.29	554	16.64	3330	3562	21.24	2847	16.98	16769	4963	25.99	2505	13.12	19095
	Yes	18	4.96	188	51.79	363	9	0.9	708	70.59	1003	15	1.43	571	54.28	1052
	Total	727	19.69	742	20.09	3693	3571	20.09	3555	20	17772	4978	24.71	3076	15.27	20147
Modern contraceptive	No	27	14.59	65	35.14	185	28	4.11	372	54.63	681	81	7.53	417	38.75	1076
	Yes	34	9.26	161	43.87	367	124	7.46	755	45.43	1662	155	7.41	714	34.15	2091
	Total	61	11.05	226	40.94	552	152	6.49	1127	48.1	2343	236	7.45	1131	35.71	3167
Information on pregnancy complication	No	185	17.19	224	20.82	1076	548	13.09	767	18.33	4185	931	22.81	485	11.88	4081

Nigeria		2003					2008					2013				
		Poorest	%	Richest	%	All women	Poorest	%	Richest	%	All women	Poorest	%	Richest	%	All women
	Yes	146	10.77	480	35.4	1356	537	8.3	2531	39.13	6468	1004	10.9	2449	26.59	9211
	Total	331	13.61	704	28.95	2432	1085	10.18	3298	30.96	10653	1935	14.56	2934	22.07	13292
Unmet need for FP	No	609	19.67	599	19.35	3096	3306	20.98	3034	19.25	15760	4609	25.65	2673	14.87	17971
	Yes	116	19.5	144	24.2	595	246	12.81	517	26.93	1920	349	16.37	401	18.81	2132
	Total	725	19.64	743	20.13	3691	3552	20.09	3551	20.08	17680	4958	24.66	3074	15.29	20103
Health worker's assistance during pregnancy outside a facility	No	622	25.99	261	10.91	2393	3324	25.97	1357	10.6	12799	4490	33.36	954	7.09	13461
	Yes	104	8.04	481	37.17	1294	233	4.74	2191	44.61	4912	469	7.06	2127	32.04	6639
	Total	726	19.69	742	20.12	3687	3557	20.08	3548	20.03	17711	4959	24.67	3081	15.33	20100
ANC: nurse assisted	No	622	25.99	261	10.91	2393	3324	25.97	1357	10.6	12799	4490	33.36	954	7.09	13461
	Yes	104	8.04	481	37.17	1294	233	4.74	2191	44.61	4912	469	7.06	2127	32.04	6639
	Total	726	19.69	742	20.12	3687	3557	20.08	3548	20.03	17711	4959	24.67	3081	15.33	20100
ANC: Govt. hospital	No	91	11.82	254	32.99	770	256	7.84	1285	39.34	3266	286	9.63	1051	35.39	2970
	Yes	245	14.64	451	26.94	1674	812	11.07	2013	27.44	7337	1634	15.84	1886	18.28	10315
	Total	336	13.75	705	28.85	2444	1068	10.07	3298	31.1	10603	1920	14.45	2937	22.11	13285
ANC: Private hospital	No	273	15.4	450	25.38	1773	907	11.28	2082	25.89	8042	1679	16.1	1847	17.71	10429
	Yes	63	9.39	255	38	671	161	6.29	1216	47.48	2561	241	8.44	1090	38.17	2856
	Total	336	13.75	705	28.85	2444	1068	10.07	3298	31.1	10603	1920	14.45	2937	22.11	13285
ANC: 1st trimester	No	258	14.45	519	29.06	1786	776	10.04	2376	30.73	7732	1396	14.38	2030	20.9	9711
	Yes	73	11.53	181	28.59	633	285	10.1	935	33.13	2822	547	14.78	920	24.86	3700
	Total	331	13.68	700	28.94	2419	1061	10.05	3311	31.37	10554	1943	14.49	2950	22	13411

Nigeria		2003					2008					2013				
		Poorest	%	Richest	%	All women	Poorest	%	Richest	%	All women	Poorest	%	Richest	%	All women
ANC: +4 tetanus injection	No	464	28.34	110	6.72	1637	2739	32.56	455	5.41	8411	3278	41.97	267	3.42	7811
	Yes	253	12.74	618	31.12	1986	808	8.8	3055	33.27	9182	1670	13.72	2776	22.81	12168
	Total	717	19.79	728	20.09	3623	3547	20.16	3510	19.95	17593	4948	24.77	3043	15.23	19979
ANC: Home	No	306	13.36	681	29.74	2290	962	9.95	3113	32.19	9670	1860	14.5	2850	22.21	12830
	Yes	30	19.48	24	15.58	154	106	11.36	185	19.83	933	60	13.19	87	19.12	455
	Total	336	13.75	705	28.85	2444	1068	10.07	3298	31.1	10603	1920	14.45	2937	22.11	13285
Home delivery	No	128	9.22	521	37.51	1389	299	4.87	2742	44.69	6135	631	8.1	2470	31.69	7794
	Yes	600	25.96	222	9.61	2311	3280	28.07	823	7.04	11684	4354	35.12	616	4.97	12398
	Total	728	19.68	743	20.08	3700	3579	20.09	3565	20.01	17819	4985	24.69	3086	15.28	20192
Delivery: government health facility	No	658	22.5	468	16.01	2924	3384	23.92	2095	14.81	14149	4549	30.14	1682	11.14	15092
	Yes	70	9.02	275	35.44	776	195	5.31	1470	40.05	3670	436	8.55	1404	27.53	5100
	Total	728	19.68	743	20.08	3700	3579	20.09	3565	20.01	17819	4985	24.69	3086	15.28	20192
Delivery: private health facility	No	671	21.67	497	16.05	3097	3483	22.49	2341	15.12	15484	4802	27.38	2025	11.54	17541
	Yes	57	9.45	246	40.8	603	96	4.11	1224	52.42	2335	183	6.9	1061	40.02	2651
	Total	728	19.68	743	20.08	3700	3579	20.09	3565	20.01	17819	4985	24.69	3086	15.28	20192
Birth assistance: Doctor	No	711	20.92	590	17.36	3399	3504	21.57	2632	16.2	16246	4832	26.86	2161	12.01	17988
	Yes	15	5.21	152	52.78	288	53	3.62	916	62.53	1465	127	6.01	920	43.56	2112
	Total	726	19.69	742	20.12	3687	3557	20.08	3548	20.03	17711	4959	24.67	3081	15.33	20100
Caesarean section	No	720	20.08	693	19.33	3586	3563	20.38	3352	19.18	17479	4948	25.29	2817	14.40	19564
	Yes	5	7.14	38	54.29	70	14	4.24	208	63.03	330	35	7.01	238	47.70	499

Nigeria	2003					2008					2013				
	Poorest	%	Richest	%	All women	Poorest	%	Richest	%	All women	Poorest	%	Richest	%	All women
Total	725	19.83	731	19.99	3656	3577	20.09	3560	19.99	17809	4983	24.84	3055	15.23	20063

Title of data: C - Concentration Indices with Covariates: Ghana (years 2000, 2005 & 2014) and Nigeria (years 2003, 2008 & 2013)
Description of data: Concentration Indices with Covariates and F-test result

	GHANA			NIGERIA		
**Service use / covariate**	**2003**	**2008**	**2014**	**2003**	**2008**	**2013**
	**CI**	**CI**	**CI**	**CI**	**CI**	**CI**
**Family planning info: health facility**	-0.01	-0.04	-0.04*	0.14*	0.14*	0.15*
Age group						
15-24	0*	0.05	-0.02	0.14	0.18*	0.18*
25-49	-0.01	-0.06	-0.05*	0.13*	0.12*	0.14*
F-test	0.09	5.58	0.84	0.23*	3.01	3.48
Marital status	-0.01	-0.04	-0.04*	0.14*	0.14*	0.15*
never	-0.02	-0.06	-0.01	0.04	0.06	0.03
Currently / previously	-0.01	-0.04	-0.04*	0.14*	0.14*	0.16*
F-test	0.85	2.08	0.00	0.01*	1.65	1.89
Maternal occupation	-0.01	-0.04	-0.04*	0.14*	0.14*	0.15*
not working	0.05	0	0	0.2	0.18*	0.21*
Professional / sales+	0	-0.04	-0.07*	0.14*	0.09*	0.12*
Agric	-0.01	-0.05	0	0.06	0.11*	0.08
others	-0.02	0.02	-0.01	0.13	0.16*	0.14*
F-test	0.52	0.49	2.96	0.52*	3.42	7.75*
Location	-0.01	-0.04	-0.04*	0.14*	0.14*	0.15*
urban	-0.02	-0.01	-0.02	0.02	0.08*	0.05*
rural	-0.02	-0.01	-0.01	0.15	0.16*	0.19*
F-test	1.66	0.04	0.00	4.98*	8.42*	39.97*
Region (Ghana)	-0.01	-0.04	-0.04*			
Western	-0.09	0.06	-0.07			
Central	0.05	0.1	-0.04			
Greater Accra	0.04	-0.01	-0.06			
Volta	0.07	0.01	0.03			
Eastern	-0.05	-0.15	-0.03			
Ashanti	0.06	0.14*	0			
Brong Ahafo	0.01	-0.03	0.01			
Northern	0.06	-0.15	-0.01			
Upper West	-0.05	0.01	0.02			
Upper East	0.07	-0.06	0.07			
F-test	1.60	2.63*	2.25			
Region (Nigeria)						
North Central				0.11	0.07	0.13*
North East				0.09	0.19*	0.1
North West				0.39*	0.29*	0.24*
South East				-0.05	0.02	0.03
South South				0.1	0.02	0.05
South West				-0.02	0.03	0
F-test				3.21*	8.64*	22.72*
**Family planning worker visit**	-0.09*	0	-0.1	0.25*	0.39*	0.4*
Age group						
15-24	0	0.1	0	0.2	0.49*	0.43*
25-49	-0.11*	0	-0.1	0.25*	0.35*	0.38*
F-test	4.5	3.8	1.9	2.5	12.88*	3
Marital status	-0.09*	0	-0.1	0.25*	0.39*	0.4*
never	-0.1	0.1	0	0	0	0
Currently / previously	-0.09*	0	-0.1	0.26*	0.4*	0.41*
F-test	0.2	1.5	0.7	1.02*	16.72*	17.47*
Maternal occupation						
not working	0.1	0.2	0	0.43*	0.51*	0.44*
Professional / sales+	-0.18*	-0.1	0	0.27*	0.32*	0.37*
Agric	-0.1	-0.14*	0	0.1	0.25*	0.2*
others	-0.2	0.1	-0.1	0.3	0.4*	0.36*
F-test	3.3	3.3	2.5	1.85*	11.74*	9.65*
Location						

Service use / covariate	GHANA 2003 CI	2008 CI	2014 CI	NIGERIA 2003 CI	2008 CI	2013 CI
urban	-0.15*	0	0	0.18*	0.2*	0.14*
rural	0	0	0	0.2	0.39*	0.42*
F-test	7.05*	0.5	0.9	0.87*	13.12*	75.99*
Region (Ghana)						
Western	-0.1	0.1	0			
Central	0	0	0			
Greater Accra	-0.2	0	-0.1			
Volta	0	0.1	-0.1			
Eastern	0	-0.1	0.1			
Ashanti	0.1	0.1	0.1			
Brong Ahafo	-0.1	0.3	0.1			
Northern	0	0	0			
Upper West	-0.2	0	-0.1			
Upper East	0	0	0.1			
F-test	1.2	1.5	1.3			
Region (Nigeria)						
North Central				0.47*	0.36*	0.33*
North East				0.3	0.34*	0.3*
North West				0.2	0.58*	0.37*
South East				0.25*	0.21*	0.1
South South				0.1	0	0
South West				0	0.16*	0.08*
F-test				0.84*	22.52*	11.55*
**Family planning: TV**	0.37*	0.4*	0.28*	0.5*	0.58*	0.56*
Age group						
15-24	0.3*	0.37*	0.26*	0.44*	0.6*	0.61*
25-49	0.39*	0.41*	0.29*	0.51*	0.56*	0.53*
F-test	10.04*	1.5	0.1	2.26*	6.6	6.4
Marital status						
never	0.2*	0.22*	0.2*	0.1	0.29*	0.31*
Currently / previously	0.37*	0.42*	0.29*	0.51*	0.59*	0.57*
F-test	7.44*	14.01*	9.22*	16.05*	33.99*	19.30*
Maternal occupation						
not working	0.33*	0.4*	0.24*	0.52*	0.64*	0.65*
Professional / sales+	0.23*	0.27*	0.15*	0.45*	0.48*	0.5*
Agric	0.32*	0.46*	0.32*	0.51*	0.51*	0.41*
others	0.27*	0.26*	0.21*	0.42*	0.51*	0.49*
F-test	3.5	7.64*	13.31*	2.23*	20.73*	16.58*
Location						
urban	0.17*	0.19*	0.13*	0.29*	0.28*	0.25*
rural	0.32*	0.44*	0.35*	0.39*	0.63*	0.63*
F-test	28.75*	55.64*	158.19*	0.95*	275.45*	140.91*
Region (Ghana)						
Western	0.22*	0.48*	0.17*			
Central	0.18*	0.26*	0.22*			
Greater Accra	0.19*	0.12*	0.05*			
Volta	0.42*	0.38*	0.25*			
Eastern	0.23*	0.32*	0.32*			
Ashanti	0.28*	0.21*	0.17*			
Brong Ahafo	0.28*	0.44*	0.4*			
Northern	0.5*	0.41*	0.37*			
Upper West	0.54*	0.67*	0.4*			
Upper East	0.4	0.55*	0.52*			
F-test	4.71*	6.48*	14.63*			
Region (Nigeria)						
North Central				0.6*	0.65*	0.56*
North East				0.46*	0.64*	0.69*

Service use / covariate	GHANA 2003 CI	2008 CI	2014 CI	NIGERIA 2003 CI	2008 CI	2013 CI
North West				0.51*	0.77*	0.76*
South East				0.3*	0.33*	0.14*
South South				0.29*	0.33*	0.23*
South West				0.29*	0.28*	0.21*
F-test				3.84*	53.47*	85.20*
**Family planning: Print**	0.54*	0.52*	0.42*	0.43*	0.65*	0.64*
Age group						
15-24	0.5*	0.56*	0.3	0.4*	0.63*	0.62*
25-49	0.55*	0.51*	0.44*	0.44*	0.64*	0.62*
F-test	0.1	0.4	3.6	0.25*	0	0
Marital status						
never	0.3	0.5	0.4	0.2	0.36*	0.3
Currently / previously	0.55*	0.52*	0.43*	0.45*	0.66*	0.65*
F-test	2.9	0	0.6	0.79*	9.88*	8.68*
Maternal occupation						
not working	0.45*	0.46*	0.1	0.42*	0.7*	0.73*
Professional / sales+	0.4*	0.43*	0.31*	0.4*	0.57*	0.56*
Agric	0.43*	0.55*	0.3	0.5	0.42*	0.5*
others	0.43*	0.37*	0.2	0.38*	0.57*	0.59*
F-test	0.2	0.5	0.2	1.33*	5.55*	4.60*
Location						
urban	0.31*	0.36*	0.26*	0.34*	0.39*	0.36*
rural	0.45*	0.54*	0.45*	0.3*	0.67*	0.67*
F-test	2.9	2.1	4.7	0.30*	42.82*	13.89*
Region (Ghana)						
Western	0.69*	0.6	0.3			
Central	0.4	0.3	0.4			
Greater Accra	0.32*	0.47*	0.1			
Volta	0.4	0.57*	0.48*			
Eastern	0.5	0.4	0.3			
Ashanti	0.41*	0.49*	0.61*			
Brong Ahafo	0.42*	0.64*	0.55*			
Northern	0.3	0.4	0.9			
Upper West	0.9	0.74*	0.5			
Upper East	0.2	0.72*	0.5			
F-test	0.4	0.8	1.7			
Region (Nigeria)						
North Central				0.57*	0.75*	0.75*
North East				0.37*	0.61*	0.71*
North West				0.32*	0.77*	0.77*
South East				0.38*	0.45*	0.24*
South South				0.25*	0.49*	0.35*
South West				0.42*	0.41*	0.45*
F-test				1.51*	12.52*	19.37*
**Modern contraceptive**	0	-0.1	-0.06*	0	-0.04*	0
Age group						
15-24	0	-0.1	-0.08*	0.1	0	0
25-49	0	0	-0.05*	0	-0.05*	0
F-test	0	0	7.25*	0.17*	5	3.2
Marital status						
never	0	-0.2	-0.1	0.1	0.1	0.07*
Currently / previously	0	0	-0.06*	0	-0.04*	0
F-test	5.2	*	*	0.05*	1.6	4.6
Maternal occupation						
not working	0	-0.1	-0.1	0.1	-0.1	0
Professional / sales+	0	0	-0.08*	0	0	0
Agric	0	-0.07*	0	0	-0.09*	0

Service use / covariate	GHANA 2003 CI	2008 CI	2014 CI	NIGERIA 2003 CI	2008 CI	2013 CI
others	0	0	-0.07*	0	0	-0.1
F-test	0.3	0.6	2.8	0.59*	4.67*	3.2
Location						
urban	0	-0.1	-0.07*	0	0	0
rural	0	-0.1	0	0	-0.04*	0
F-test	5.23*	0.35*	0.01*	0.00*	1.9	6.69*
Region (Ghana)						
Western	0	-0.1	0			
Central	0.1	-0.1	0			
Greater Accra	-0.1	0	-0.11*			
Volta	0	0	0			
Eastern	0	0.1	0			
Ashanti	0	0	-0.09*			
Brong Ahafo	0	0	0			
Northern	0.19*	0	0			
Upper West	0	0	0			
Upper East	0	0	0			
F-test	2.1	1.2	6.69*			
Region (Nigeria)						
North Central				0	0	0
North East				-0.1	0	0
North West				0.15*	0	0.07*
South East				0.23*	-0.1	-0.1
South South				0	0	0
South West				0	0	0
F-test				10.23*	1	2.5
**Information on pregnancy complication**	0.08*	0.07*	0.02*	0.13*	0.12*	0.1*
Age group						
15-24	0.06*	0.06*	0	0.16*	0.12*	0.1*
25-49	0.09*	0.07*	0.02*	0.11*	0.11*	0.09*
F-test	2.8	0.4	1	6.72*	0.3	0
Marital status						
Never	0.15*	0.1	0	0.1	0.1	0.08*
Currently / previously	0.08*	0.07*	0.02*	0.13*	0.12*	0.1*
F-test	0.2	0.3	0.9	4.09*	6	0.9
Maternal occupation						
not working	0.15*	0	0	0.17*	0.15*	0.1*
Professional / sales+	0.06*	0.04*	0	0.11*	0.09*	0.09*
Agric	0.1	0.06*	0	0.1	0	0.07*
others	0.1	0	0.03*	0.11*	0.11*	0.08*
F-test	2.2	0.1	6.41*	0.89*	23.48*	1.8
Location						
urban	0	0	0.02*	0.08*	0.07*	0.05*
rural	0.07*	0.07*	0	0.09*	0.1*	0.08*
F-test	6.2	8.83*	1.2	1.85*	11.85*	3.3
Region (Ghana)						
Western	0.12*	0.09*	0			
Central	0.1	0.1	0			
Greater Accra	0.08*	0	0			
Volta	0.1	0	0			
Eastern	0.09*	0	0.1			
Ashanti	0	0.07*	0.1			
Brong Ahafo	0.06*	0.1*	0			
Northern	0	0.1	0			
Upper West	0.1	0.1	0.03*			
Upper East	0.1	0	-0.1			

Service use / covariate	GHANA 2003 CI	2008 CI	2014 CI	NIGERIA 2003 CI	2008 CI	2013 CI
F-test	1.7	2.48*	3.71*			
Region (Nigeria)						
North Central				0.11*	0.2*	0.09*
North East				0.1	0.07*	0
North West				0.12*	0.09*	0.08*
South East				0.17*	0.11*	0
South South				0.16*	0.07*	0
South West				0	0.03*	0.05*
F-test				7.17*	17.87*	4.13*
**Family planning: unmet need**	0	0	0	0.1	0.18*	0.18*
Age group						
15-24	0.08*	0.1	0.1	0.1	0.25*	0.22*
25-49	0	0	0	0	0.17*	0.17*
F-test	11.00*	5.2	4.1	1.77*	4.6	0.5
Marital status						
never	0	0.09*	0	0	0	0
Currently / previously	0	0	0	0.1	0.19*	0.19*
F-test	1.6	1.2	0.5	0.08*	3.5	1.6
Maternal occupation						
not working	0.1	0	0	0.1	0.21*	0.29*
Professional / sales+	0	-0.09*	-0.11*	0.1	0.16*	0.21*
Agric	0.07*	0.09*	0.1	0.1	0.12*	0
others	0	-0.1	-0.1	0.1	0.3*	0.19*
F-test	6.42*	9.19*	8.22*	0.21*	4.41*	5.16*
Location						
urban	0	-0.1	-0.11*	0.1	0	0
rural	0.07*	0.07*	0.1	0	0.22*	0.21*
F-test	24.59*	25.55*	31.12*	0.00*	33.55*	20.63*
Region (Ghana)						
Western	0	-0.1	-0.17*			
Central	-0.1	-0.1*	-0.12*			
Greater Accra	-0.14*	-0.1	-0.1			
Volta	0	-0.1	-0.1			
Eastern	0	0	-0.1			
Ashanti	-0.1	-0.1	-0.1*			
Brong Ahafo	-0.1	0	-0.12*			
Northern	0	0	0			
Upper West	0	0	0.1			
Upper East	0	-0.1	0			
F-test	1.2	0.9	0.9			
Region (Nigeria)						
North Central				0	0	-0.1
North East				0.17*	0.1	0.1
North West				0	0.21*	0.2
South East				0.22*	0.1	-0.2*
South South				-0.1	0	-0.1
South West				0	0	0
F-test				2.27*	4.22*	6.36*

$p \leq 0.01$

+Professional, technical, managerial, Clerical, or sales

Title of data: D – Concentration Indices with Covariates: Ghana (years 2000, 2005 & 2014) and Nigeria (years 2003, 2008 & 2013)
Description of data: Concentration Indices with Covariates and F-test result

Service use / covariate	GHANA			NIGERIA		
	2003	2008	2014	2003	2008	2013
	CI	CI	CI	CI	CI	CI
**Health worker's assistance during pregnancy outside a facility**	-0.25*	-0.21*	-0.27*	-0.17*	-0.21*	-0.21*
Age group						
15-24	-0.23*	-0.06	-0.19*	-0.13*	-0.14*	-0.15*
25-49	-0.26*	-0.25*	-0.29*	-0.19*	-0.23*	-0.23*
F-test	1.23	18.51*	1.78	12.16*	128.66*	81.64*
Marital status						
never	-0.21*	-0.2	-0.2	-0.23	-0.15*	-0.17*
Currently / previously	-0.25*	-0.21*	-0.28*	-0.17*	-0.21*	-0.21*
F-test	0.02	0.21	0.00	0.00	25.08*	1.01
Maternal occupation						
not working	-0.29*	-0.32*	-0.2*	-0.16*	-0.17*	-0.17*
Professional / sales+	-0.35*	-0.2*	-0.29*	-0.19*	-0.28*	-0.26*
Agric	-0.07*	-0.03	-0.08	-0.14*	-0.09*	-0.1*
others	-0.29*	-0.24*	-0.23*	-0.18*	-0.23*	-0.23*
F-test	46.17*	10.32*	4.52*	5.17*	166.75*	71.41*
Location						
urban	-0.3*	-0.18*	-0.19*	-0.22*	-0.27*	-0.24*
rural	-0.09*	-0.07*	-0.17*	-0.1*	-0.13*	-0.11*
F-test	86.48*	10.81*	0.06	29.1*	268.66*	102.28*
Region (Ghana)						
Western	-0.16*	-0.29*	-0.32*			
Central	-0.08	-0.19*	0.05			
Greater Accra	-0.48*	-0.14	-0.37*			
Volta	-0.14*	-0.02	-0.07			
Eastern	-0.14*	-0.15*	-0.21*			
Ashanti	-0.33*	-0.33*	-0.34			
Brong Ahafo	-0.26*	-0.19*	-0.08			
Northern	-0.05*	-0.1	-0.16*			
Upper West	-0.13*	-0.15	-0.31*			
Upper East	-0.12*	-0.16*	-0.02			
F-test	12.3*	3.5*	4.53*			
Region (Nigeria)						
North Central				-0.21*	-0.15*	-0.19*
North East				-0.08*	-0.08*	-0.11*
North West				-0.08*	-0.06*	-0.08*
South East				-0.65*	-0.27*	-0.29*
South South				-0.17*	-0.21*	-0.2*
South West				-0.26*	-0.24*	-0.23*
F-test				16.92*	67.68*	37.12*
**ANC: nurse assisted**	0.29*	0.24*	0.11*	0.33*	0.4*	0.39*
Age group						
15-24	0.26*	0.19*	0.11*	0.32*	0.4*	0.39*
25-49	0.3*	0.25*	0.11*	0.33*	0.39*	0.39*
F-test	1.81	4.75	0.5	0.91	0.33	0.94
Marital status						
never	0.1	0.11*	0.09*	0.15	0.23*	0.16*
Currently / previously	0.29*	0.24*	0.11*	0.34*	0.4*	0.4*
F-test	11.14*	8.06*	4.96	4.84	23.14*	21.95*
Maternal occupation						
not working	0.26*	0.2*	0.08*	0.46*	0.49*	0.47*
Professional / sales+	0.16*	0.14*	0.04*	0.3*	0.35*	0.38*
Agric	0.21*	0.26*	0.12*	0.28*	0.26*	0.2*

Service use / covariate	GHANA 2003 CI	2008 CI	2014 CI	NIGERIA 2003 CI	2008 CI	2013 CI
others	0.21*	0.14*	0.08*	0.29*	0.4*	0.38*
F-test	2.19	7.44*	4.33*	10.73*	42.92*	41.97*
Location						
urban	0.06*	0.06*	0.02	0.16*	0.17*	0.12*
rural	0.24*	0.24*	0.12*	0.31*	0.41*	0.43*
F-test	40.24*	57.45*	53.44*	14.34*	267.42*	380.74*
Region (Ghana)						
Western	0.28*	0.21*	0.08*			
Central	0.24*	0.26*	0.1*			
Greater Accra	0.09	0.09*	0.05			
Volta	0.31*	0.22*	0.13*			
Eastern	0.17*	0.18*	0.14*			
Ashanti	0.17*	0.12*	0.01			
Brong Ahafo	0.23*	0.18*	0.09*			
Northern	0.31*	0.33*	0.19*			
Upper West	0.25*	0.27*	0.06*			
Upper East	0.36*	0.25*	0.14			
F-test	3.67*	5.28*	6.55*			
Region (Nigeria)						
North Central				0.21*	0.28*	0.27*
North East				0.44*	0.53*	0.5*
North West				0.55*	0.58*	0.51*
South East				0.1*	0.13*	0.09*
South South				0.2*	0.2*	0.19*
South West				0.05	0.1*	0.07*
F-test				12.36*	83.28*	106.45*
**ANC: government health facility**	-0.02*	-0.03*	-0.04*	-0.04	-0.07*	-0.07*
Age group						
15-24	-0.01	-0.01	-0.02	-0.04	-0.05*	-0.06*
25-49	-0.02*	-0.04*	-0.05*	-0.04	-0.07*	-0.07*
F-test	2.33	5.5	2.9	0.32	2.3	1.65
Marital status						
never	-0.08*	0.02	0	-0.15	-0.03	-0.05
Currently / previously	-0.02*	-0.03*	-0.04*	-0.04	-0.07*	-0.07*
F-test	19.9*	1.91	0.98	4.56	3.02	2.4
Maternal occupation						
not working	-0.01	-0.04*	-0.03*	-0.02	-0.08*	-0.08*
Professional / sales+	-0.02	-0.02	-0.05*	-0.05*	-0.08*	-0.08*
Agric	0	-0.01	0	-0.06	0	-0.01
others	-0.03	-0.05*	-0.02	-0.07	-0.1*	-0.09*
F-test	3.96*	4.62*	7.99*	1.06	14.68*	8.96*
Location						
urban	-0.03*	-0.01	-0.04*	-0.03	-0.1*	-0.07*
rural	0	-0.02*	-0.02*	-0.03	-0.03*	-0.03*
F-test	39.48*	2.62	5.66	0	91.01*	23*
Region (Ghana)						
Western	-0.02	-0.01	-0.02			
Central	0.02	-0.02	-0.03			
Greater Accra	-0.03	-0.01	-0.07*			
Volta	-0.01	-0.03	0.02			
Eastern	0.01	0.02	-0.02			
Ashanti	-0.02	-0.04*	-0.05*			
Brong Ahafo	0.01	0.02	-0.01			

Service use / covariate	GHANA 2003 CI	2008 CI	2014 CI	NIGERIA 2003 CI	2008 CI	2013 CI
Northern	-0.01	-0.02	0			
Upper West	0.01	0	-0.02			
Upper East	0.04	-0.01	0			
F-test	2.61*	4.95*	8.66*			
Region (Nigeria)						
North Central				-0.04	0.03	-0.02
North East				-0.01	0.01	0
North West				0.01	-0.02*	0
South East				-0.12	-0.05	-0.11*
South South				0.05	0.01	-0.02
South West				-0.11*	-0.08*	-0.08*
F-test				4.82*	19.02*	28.48*
**ANC: Private health facility**	0.24*	0.3*	0.36*	0.2*	0.23*	0.27*
Age group						
15-24	0.15	0.05	0.21	0.23*	0.21*	0.27*
25-49	0.27*	0.34*	0.38*	0.19*	0.23*	0.25*
F-test	1.71	4.37	4.68	0.25*	0.42	0.14
Marital status						
never	0.44*	-0.10	0.26	0.37*	0.17	0.17*
Currently / previously	0.23*	0.33*	0.36*	0.19*	0.23*	0.27*
F-test	1.7	4.87	0.78	1.89*	2.34	2.68
Maternal occupation						
not working	0.12	0.42*	0.27*	0.24*	0.35*	0.37*
Professional / sales+	0.17*	0.16	0.32*	0.18*	0.23*	0.27*
Agric	0.15	0.12	-0.08	0.2	0.01	0.01
others	0.19*	0.32*	0.27*	0.24*	0.26*	0.33*
F-test	0.31	1.58	10.61*	1.12*	50.81*	26.56*
Location						
urban	0.17*	0.11	0.26*	0.14*	0.21*	0.17*
rural	0.09	0.32*	0.26*	0.18	0.15*	0.21*
F-test	1.29	7.56*	0.22	0.05*	15.45*	1.69
Region (Ghana)						
Western	0.22	0.18	0.14			
Central	-0.07	0.22	0.36*			
Greater Accra	0.12	0.27*	0.28*			
Volta	0.3	0.27	-0.16			
Eastern	0.12	0	0.11			
Ashanti	0.13	0.23*	0.33*			
Brong Ahafo	0.34*	-0.06	0.13			
Northern	0.31	0.44	0.15			
Upper West	-0.16	-0.43	0.48			
Upper East	-0.19	0.56	0.71			
F-test	1.39	1.97	0.9			
Region (Nigeria)						
North Central				0.11	0.01	0.09
North East				0.2	0.11	0.01
North West				0	0.3*	0.14
South East				0.13*	0.12*	0.13*
South South				0.21	0.27*	0.22*
South West				0.13*	0.14*	0.11*
F-test				4.15*	8.98*	3.65*
**ANC: 1st trimester**	0.09*	0.09*	0.07*	-0.01	0.03	0.04
Age group						

Service use / covariate	GHANA 2003 CI	2008 CI	2014 CI	NIGERIA 2003 CI	2008 CI	2013 CI
15-24	0.08*	0.08	0.05	0.05	-0.03	0.01
25-49	0.09*	0.09*	0.07*	-0.03	0.04*	0.04
F-test	0.9	0.34	2.55	3.68*	18*	6.07
Marital status						
never	0.03	0.01	0.08*	0.24	-0.06	0.02
Currently / previously	0.09*	0.1*	0.07*	-0.02	0.03	0.04
F-test	2.02	1.95	0.15	3.23*	1.33	1.39
Maternal occupation						
not working	0.13*	0.1*	0.08*	0.07	0.02	0.06
Professional / sales+	0.07*	0.07*	0.05*	-0.05	0.06*	0.04
Agric	0.06*	0.06	0.02	0	0.01	-0.06
others	0.04	0.04	0.05*	-0.03	0.1*	0.11*
F-test	0.9	1	5.25*	3.17*	3.55*	6.45*
Location						
urban	0.06*	0.08*	0.07*	0.07	0.1*	0.09*
rural	0.06*	0.08*	0.07*	-0.06	0.01	0.04*
F-test	0.96	0.25	0.13	11.63*	35.42*	6.19
Region (Ghana)						
Western	0.11	0.07	0.04			
Central	0.17*	0.04	0.08*			
Greater Accra	0.08	0.08*	0.08*			
Volta	0.04	0.02	0.05			
Eastern	0.12	-0.03	0.04			
Ashanti	0.09*	0.09*	0.07*			
Brong Ahafo	0.12*	0.04	0.07*			
Northern	0.13	0.18*	0.04			
Upper West	-0.05	0	0.01			
Upper East	-0.03	-0.02	0.01			
F-test	1.53	3.1*	3.82*			
Region (Nigeria)						
North Central				-0.01	0.07*	-0.01
North East				-0.12	-0.07	-0.08
North West				-0.19*	-0.21*	-0.16*
South East				0.23	0.1*	0.07*
South South				0.1	0.07	0.04
South West				0.00	0.1*	0.07
F-test				6.23*	39.75*	28.43*
**ANC: +4 tetanus injection**	0.05*	0.03*	0.02*	0.23*	0.27*	0.22*
Age group						
15-24	0.05*	0.04*	0.02*	0.24*	0.28*	0.22*
25-49	0.05*	0.03*	0.02*	0.23*	0.25*	0.22*
F-test	0.00	1.78	0.02	0.01*	5.75	0.00
Marital status						
never	0.01	0.07*	0.02	0.08	0.1*	0.07*
Currently / previously	0.05*	0.03*	0.02*	0.24*	0.27*	0.23*
F-test	7.18*	1.05	0.31	9.22*	47.96*	29.26*
Maternal occupation						
not working	0.04	0.06*	0.01	0.33*	0.35*	0.29*
Professional / sales+	0.02*	0.02*	0.01	0.24*	0.23*	0.21*
Agric	0.05*	0.02	0.01	0.14*	0.18*	0.1*
others	0.03*	0.03*	0.01	0.21*	0.27*	0.19*
F-test	3.9*	2.86	2.45	9.95*	79.63*	86.53*
Location						
urban	0.01	0.02*	0.01	0.11*	0.09*	0.06*

Service use / covariate	GHANA 2003 CI	2008 CI	2014 CI	NIGERIA 2003 CI	2008 CI	2013 CI
rural	0.03*	0.03*	0.02*	0.2*	0.28*	0.23*
F-test	21.76*	7.75*	0.03	6.34*	437.41*	300.26*
Region (Ghana)						
Western	0.01	0.03	0.02			
Central	0.02	0.02	-0.01			
Greater Accra	0.04*	0.05*	0.01			
Volta	0.08*	0.01	0.01			
Eastern	0.00	0.03	0.02			
Ashanti	0.04*	0.02	0.01			
Brong Ahafo	0.04*	0.05*	0.01			
Northern	0.05	0.05*	0.01			
Upper West	0.03	0.00	0.01			
Upper East	0.04	-0.02	0.03			
F-test	2.66*	4.58*	2.17			
Region (Nigeria)				0.23*	0.27*	0.22*
North Central				0.15*	0.16*	0.14*
North East				0.24*	0.3*	0.2*
North West				0.43*	0.4*	0.29*
South East				0.04*	0.06*	0.02*
South South				0.13*	0.11*	0.07*
South West				0.02	0.06*	0.08*
F-test				25.79*	205.75*	121.51*
**ANC: Home**	-0.26*	-0.25*	-0.1	-0.23*	-0.15*	0.05
Age group						
15-24	-0.07	-0.23	0.15	-0.23	-0.08	0.21*
25-49	-0.3*	-0.26	-0.19	-0.24*	-0.17*	0.01
F-test	0.79	0.26	0.6	0.01*	1.56	5.74
Marital status						
never	0.08		-0.34	-0.31	-0.08	-0.02
Currently / previously	-0.27*		-0.07	-0.22*	-0.16*	0.06
F-test	0.81		1.37	0*	1.02	0.29
Maternal occupation						
not working	-0.22		0.21	-0.33	-0.22*	0.05
Professional / sales+	-0.27		-0.13	-0.22	-0.14*	0.09
Agric	-0.24		-0.06	0.05	0.01	0.11
others	-0.43		-0.53	-0.24	-0.07	0.13
F-test	0.20		1.24	2.17*	6.27*	0.51
Location						
urban	-0.41		-0.18	-0.29*	-0.13	-0.02
rural	-0.1		0.03	-0.12	-0.09*	0.11
F-test	2.55		1.31	3.93*	0.02	3.54
Region (Ghana)						
Western	-0.29		0.35			
Central	-0.74		0.63			
Greater Accra	-0.86		-0.83			
Volta	-0.21		-0.27			
Eastern	-0.02		*			
Ashanti	0.06		*			
Brong Ahafo	-0.59*		*			
Northern	-0.04		*			
Upper West	-0.23		*			
Upper East	0.25		*			
F-test	1.18		*			
Region (Nigeria)						

Service use / covariate	GHANA 2003 CI	2008 CI	2014 CI	NIGERIA 2003 CI	2008 CI	2013 CI
North Central				0.08	-0.22*	0.04
North East				0.03	-0.31*	-0.41
North West				-0.48	-0.08	-0.14
South East				-0.54*	-0.39*	-0.23*
South South				-0.26*	-0.39*	-0.21*
South West				-0.17	-0.12	-0.07
F-test				1.93*	5.23*	1.13

**p ≤ 0.01*

Title of data: E – Concentration Indices with Covariates: Ghana (years 2000, 2005 & 2014) and Nigeria (years 2003, 2008 & 2013)
Description of data: Concentration Indices with Covariates and F-test result

Service use / covariate	GHANA			NIGERIA		
	2003	2008	2014	2003	2008	2013
	CI	CI	CI	CI	CI	CI
**Delivery: home**	-0.14*	-0.13*	-0.1*	-0.12*	-0.15*	-0.14*
Age group						
15-24	-0.13*	-0.09*	-0.07*	-0.09*	-0.12*	-0.11*
25-49	-0.15*	-0.14*	-0.1*	-0.12*	-0.16*	-0.15*
F-test	1.78	15.83*	11.87*	11.31*	96.56*	51.47*
Marital status						
never	-0.1*	-0.1*	-0.07*	-0.09*	-0.09*	-0.09*
Currently / previously	-0.14*	-0.13*	-0.1*	-0.11*	-0.15*	-0.14*
F-test	0.00	0.13	2.79	0.04*	33.77*	5.48
Maternal occupation						
not working	-0.14*	-0.11*	-0.07*	-0.11*	-0.14*	-0.13*
Professional / sales+	-0.12*	-0.09*	-0.06*	-0.13*	-0.17*	-0.16*
Agric	-0.06*	-0.09*	-0.06*	-0.08*	-0.08*	-0.06*
others	-0.13*	-0.1*	-0.08*	-0.11*	-0.17*	-0.15*
F-test	33.21*	3.30	3.8*	8.45*	111.48*	70.65*
Location						
urban	-0.07*	-0.05*	-0.04*	-0.09*	-0.12*	-0.09*
rural	-0.07*	-0.1*	-0.07*	-0.08*	-0.11*	-0.09*
F-test	24.9*	2.13	0.99	17.46*	44.37*	11.49*
Region (Ghana)						
Western	-0.09*	-0.12*	-0.08*			
Central	-0.09*	-0.1*	-0.09*			
Greater Accra	-0.09*	-0.05*	-0.03			
Volta	-0.14*	-0.11*	-0.09*			
Eastern	-0.08*	-0.08*	-0.1*			
Ashanti	-0.12*	-0.09*	-0.05*			
Brong Ahafo	-0.12*	-0.1*	-0.08*			
Northern	-0.06*	-0.1*	-0.06			
Upper West	-0.08*	-0.1*	-0.05*			
Upper East	-0.09	-0.11*	-0.07			
F-test	4.21*	0.69	5.54*			
Region (Nigeria)						
North Central				-0.1*	-0.11*	-0.1*
North East				-0.07*	-0.07*	-0.08*
North West				-0.06*	-0.06*	-0.06*
South East				-0.09*	-0.1*	-0.08*
South South				-0.11*	-0.12*	-0.1*
South West				-0.06*	-0.08*	-0.07*
F-test				9.06*	75.71*	18.24*
**Delivery: government health facility**	0.10*	0.10*	0.06*	0.05*	0.07*	0.08*
Age group						
15-24	0.08*	0.07*	0.06*	0.04*	0.06*	0.07*
25-49	0.11*	0.11*	0.07*	0.06*	0.08*	0.08*
F-test	4.21	7.79*	3.76			
Marital status						
never	0.04	0.1*	0.06*	0.01	0.03	0.03
Currently / previously	0.11*	0.1*	0.07*	0.05*	0.07*	0.08*
F-test	7.4*	0.24	3.46	3.79*	14.18*	1.41
Maternal occupation						
not working	0.08*	0.06*	0.04*	0.06*	0.07*	0.08*

Service use / covariate	GHANA 2003 CI	GHANA 2008 CI	GHANA 2014 CI	NIGERIA 2003 CI	NIGERIA 2008 CI	NIGERIA 2013 CI
Professional / sales+	0.09*	0.07*	0.03*	0.05*	0.08*	0.08*
Agric	0.04*	0.07*	0.06*	0.03	0.05*	0.04*
others	0.11*	0.07*	0.06*	0.05*	0.07*	0.07*
F-test	12.19*	0.17	2.35	1.52*	8.06*	9.61*
Location	*	*	*		*	*
urban	0.04*	0.03*	0.01	0.03	0.03*	0.03*
rural	0.05*	0.08*	0.06*	0.03*	0.06*	0.06*
F-test	0.33	12.74*	4.45	3.49*	57.12*	33.84*
Region (Ghana)						
Western	0.06	0.11*	0.07*			
Central	0.05	0.09*	0.05			
Greater Accra	0.07*	0.05	0.01			
Volta	0.12*	0.09*	0.08*			
Eastern	0.08*	0.09*	0.08*			
Ashanti	0.08*	0.06*	0.00			
Brong Ahafo	0.1*	0.11*	0.08*			
Northern	0.05*	0.09*	0.06			
Upper West	0.07*	0.10*	0.02			
Upper East	0.07	0.11*	0.07			
F-test	3.59*	1.77	5.39*			
Region (Nigeria)						
North Central				0.04	0.08*	0.07*
North East				0.06*	0.07*	0.08*
North West				0.05*	0.05*	0.06*
South East				-0.02	0.01	0.00
South South				0.06*	0.05*	0.06*
South West				-0.01	0.00	0.00
F-test				6.73*	53.34*	48.29*
**Delivery: private health facility**	0.04*	0.03*	0.03*	0.06*	0.08*	0.07*
Age group						
15-24	0.04*	0.02	0.01	0.05*	0.05*	0.05*
25-49	0.04*	0.03*	0.03*	0.07*	0.09*	0.07*
F-test	1.38	1.84	6.56	8.42*	112.3*	74.21*
Marital status						
never	0.06	0.00	0.01	0.09*	0.06*	0.05*
Currently / previously	0.04*	0.03*	0.03*	0.06*	0.08*	0.07*
F-test	3.67	0.83	1.39	15.92*	1.81	0.01
Maternal occupation						
not working	0.06*	0.06*	0.03	0.06*	0.06*	0.05*
Professional / sales+	0.03*	0.02	0.04*	0.07*	0.09*	0.08*
Agric	0.01*	0.02*	0.00	0.06*	0.03*	0.01
others	0.02*	0.02	0.02	0.06*	0.09*	0.07*
F-test	9.29*	3.92*	18.65*	1.44*	110.13*	81.13*
Location						
urban	0.03	0.01	0.03*	0.06*	0.09*	0.06*
rural	0.01*	0.02*	0.01	0.04*	0.04*	0.03*
F-test	9.31*	0.11	45.85*	42.77*	477.31*	352.89*
Region (Ghana)						
Western	0.03	0.02	0.00			
Central	0.03	0.01	0.04			
Greater Accra	0.02	0.00	0.02			
Volta	0.02	0.02	0.00			
Eastern	0.00	-0.01	0.02			

Service use / covariate	GHANA 2003 CI	2008 CI	2014 CI	NIGERIA 2003 CI	2008 CI	2013 CI
Ashanti	0.04*	0.03*	0.05*			
Brong Ahafo	0.02	-0.01	0.01			
Northern	0.01	0.01	0.00			
Upper West	0.01	0.00	0.02			
Upper East	0.02	0.00	0.00			
F-test	2.45*	1.44	5.2*			
Region (Nigeria)						
North Central				0.06*	0.03*	0.03*
North East				0.01	0.01*	0*
North West				0.01	0.01*	0*
South East				0.12*	0.08*	0.08*
South South				0.06*	0.06*	0.05*
South West				0.08*	0.08*	0.07*
F-test				92.29*	205.28*	269.21*
**Birth assistance: Doctor**	0.03*	0.04*	0.05*	0.04*	0.06*	0.05*
Age group						
15-24	0.03*	0.01	0.02	0.03*	0.03*	0.03*
25-49	0.04*	0.05*	0.06*	0.04*	0.06*	0.06*
F-test	1.92	16.94*	39.72*	0.13*	181.82*	116.56*
Marital status						
never	0.06	0.03	0.04*	0.11*	0.03*	0.03*
Currently / previously	0.03*	0.04*	0.05*	0.03*	0.06*	0.05*
F-test	1.93	0.00	1.36	28.98*	10.47*	2.13
Maternal occupation						
not working	0.05*	0.06*	0.03*	0.03*	0.05*	0.04*
Professional / sales+	0.04*	0.04*	0.06*	0.05*	0.07*	0.06*
Agric	0.01*	0.01	0.01	0.04	0.01*	0.02*
others	0.04*	0.04*	0.06*	0.03*	0.06*	0.05*
F-test	14.4*	8.75*	27.89*	3.08*	112.34*	41.88*
Location						
urban	0.04*	0.04*	0.05*	0.05*	0.08*	0.07*
rural	0.01	0.02*	0.03*	0.01*	0.02*	0.02*
F-test	46.54*	9.84*	38.52*	94.02*	870.73*	423.99*
Region (Ghana)						
Western	0.01	0.04	0.02			
Central	0.00	0.00	0.03*			
Greater Accra	0.05*	0.04*	0.02			
Volta	0.03*	-0.01	0.02			
Eastern	0.02	-0.02	0.02			
Ashanti	0.03*	0.05*	0.06*			
Brong Ahafo	0.01	0.03	0.02			
Northern	0.01	0.01	0.01			
Upper West	0.01	0.02	0.02			
Upper East	0.01	0.03	0.01			
F-test	3.41*	4.22*	4.29*			
Region (Nigeria)						
North Central				0.03*	0.04*	0.03*
North East				0.01*	0.01*	0.02*
North West				0.00	0.02*	0.02*
South East				0.07*	0.06*	0.04*
South South				0.05*	0.06*	0.06*
South West				0.06*	0.09*	0.06*
F-test				31.24*	161.08*	80.27*

Service use / covariate	GHANA 2003 CI	2008 CI	2014 CI	NIGERIA 2003 CI	2008 CI	2013 CI
**C Section**	0.45*	0.3*	0.31*	0.49*	0.58*	0.49*
Age group						
15-24	0.37	0.05	0.07	0.22	0.46*	0.33*
25-49	0.47*	0.34*	0.32*	0.54*	0.59*	0.52*
F-test	0.1	4.51	20.73*	8.32*	3.47	9.4*
Marital status						
never	0.38	0.26	0.2	0.53	0.57*	0.27
Currently / previously	0.45*	0.31*	0.32*	0.48*	0.58*	0.5*
F-test	0	0.58	7.89*	0.02	0.03	1.95
Maternal occupation						
not working	0.36	0.26	0.15	0.5*	0.6*	0.59*
Professional / sales+	0.45*	0.19*	0.28*	0.44*	0.55*	0.49*
Agric	0.14	0.28	0.11	0.61	0.19	0.28
others	0.43	0.25	0.27*	0.45	0.46*	0.43*
F-test	1.29	0.37	3.03	0.66	7.04*	1.19
Location						
urban	0.36*	0.11	0.19*	0.36*	0.41*	0.36*
rural	0.14	0.38*	0.3*	0.29	0.52*	0.37*
F-test	2.32	7.19*	4.75	1.04	2.07	0.13
Region (Ghana)						
Western	0.68	0.4	0.18*			
Central	0.14	-0.05	0.24*			
Greater Accra	0.22	0.16	0.17*			
Volta	0.57	0.19	0.15			
Eastern	0.43	-0.09	0.16			
Ashanti	0.25	0.26	0.26*			
Brong Ahafo	0.14	0.36	0.12			
Northern	0.37	0.49	0.36			
Upper West	0.18	0.94	0.27			
Upper East	0.95	0.24	0.24			
F-test	0.92	2.25	0.66			
Region (Nigeria)						
North Central				0.09	0.43*	0.5*
North East				0.3	0.53	0.44*
North West				0.43	0.4	0.22
South East				0.24*	0.45*	0.14
South South				0.49	0.49*	0.39*
South West				0.6*	0.39*	0.35*
F-test				0.78	0.28	1.47

$p \leq 0.01$
