# Supplementary figures and images for: Examining trends in inequality in the use of reproductive health care services in Ghana and Nigeria
Source: BMC Pregnancy Childbirth. 2018 Dec 13;18:492. doi: 10.1186/s12884-018-2102-9 (PMC6293518; doi:10.1186/s12884-018-2102-9)

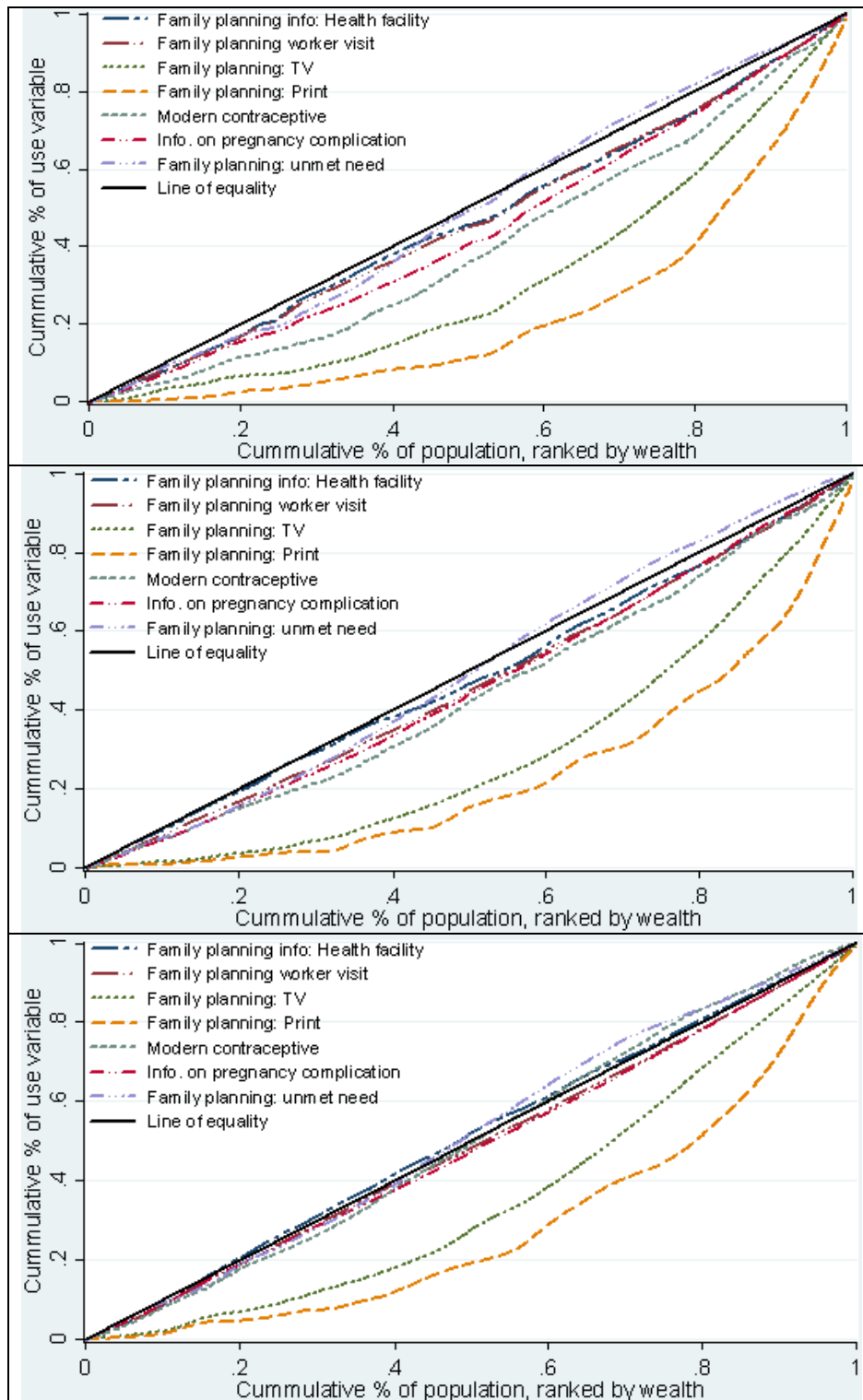

Supplement: Supplementary file 2 — Figure S1. Concentration curves of use of family planning Ghana (Years 2003, 2008, 2014). (PDF 26 kb) [file 12884_2018_2102_MOESM2_ESM.pdf]

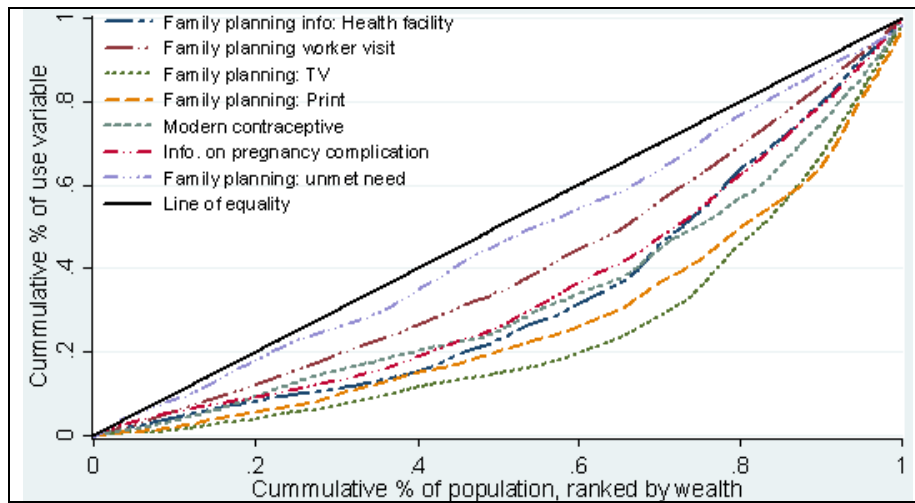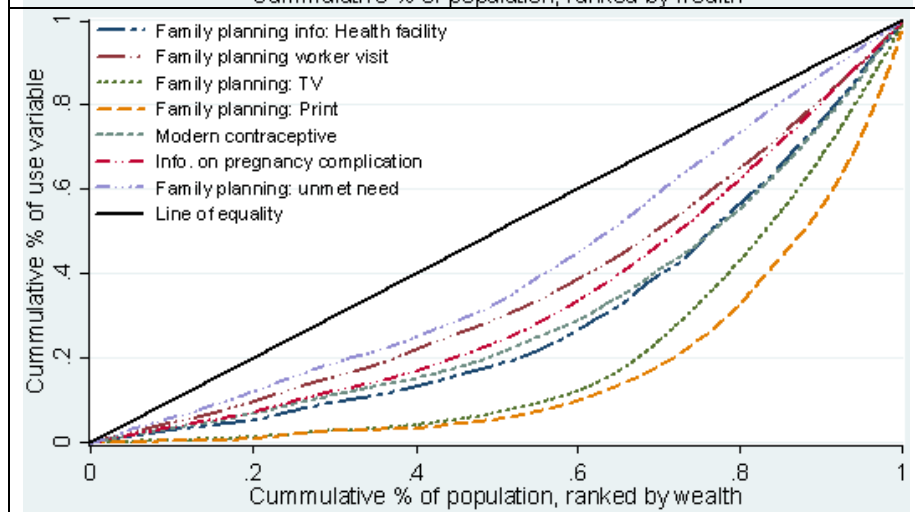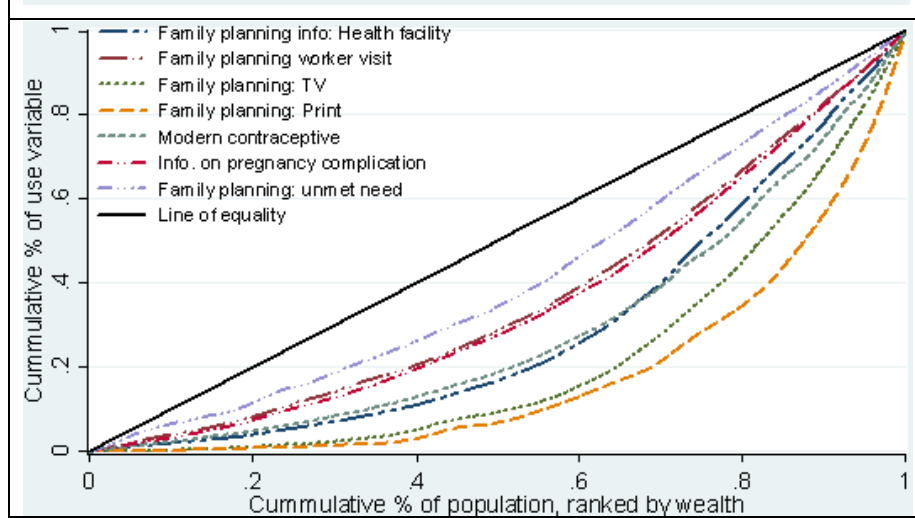

Supplement: Supplementary file 3 — Figure S2. Concentration curves of use of family planning Nigeria (Years 2003, 2008, 2013). (PDF 26 kb) [file 12884_2018_2102_MOESM3_ESM.pdf]

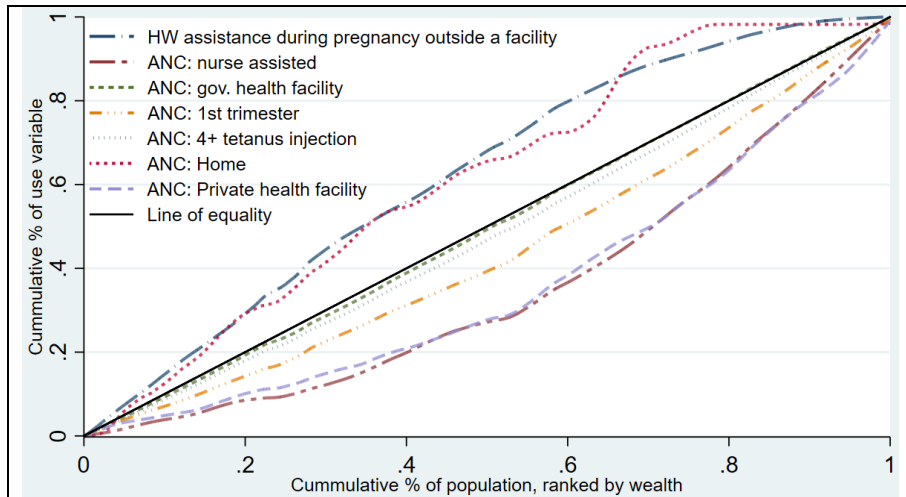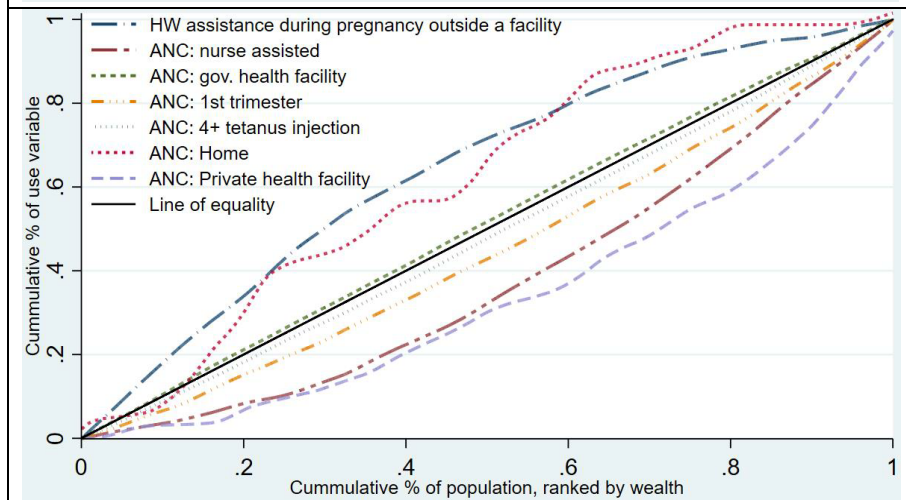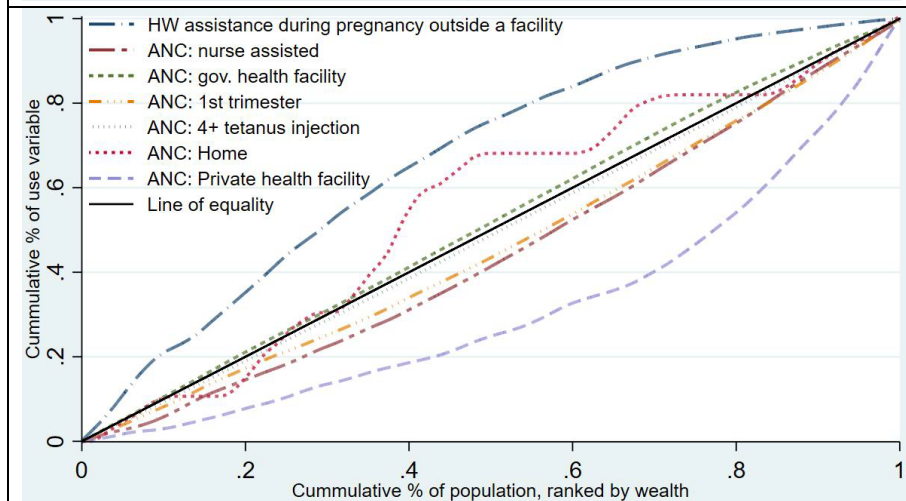

Supplement: Supplementary file 4 — Figure S3. Concentration curves of use of Antenatal care, Ghana (Years 2003, 2008, 2013). (PDF 924 kb) [file 12884_2018_2102_MOESM4_ESM.pdf]

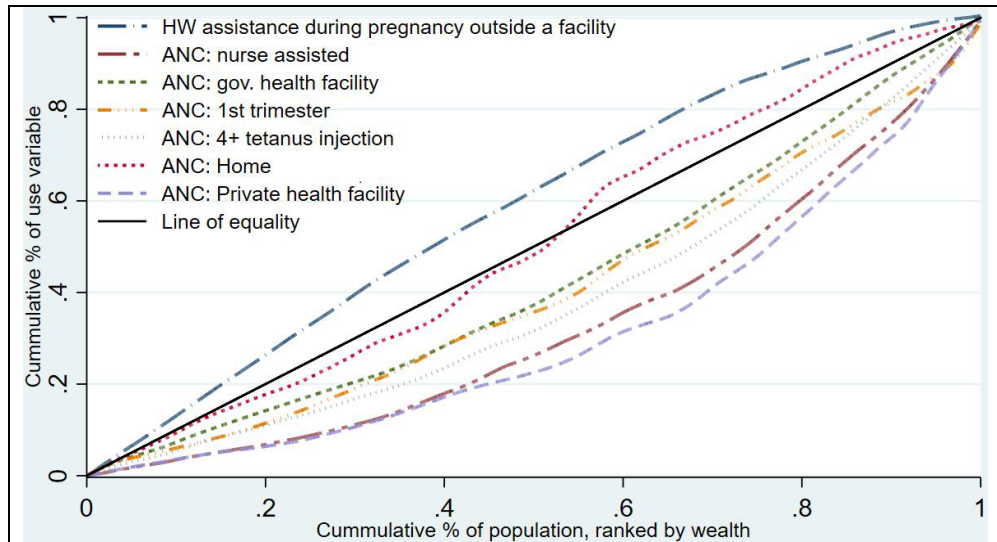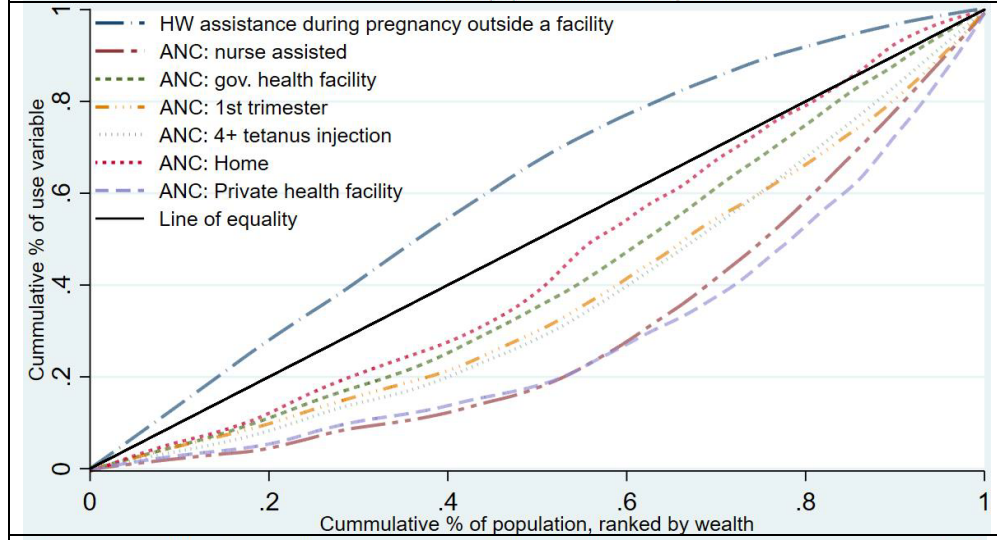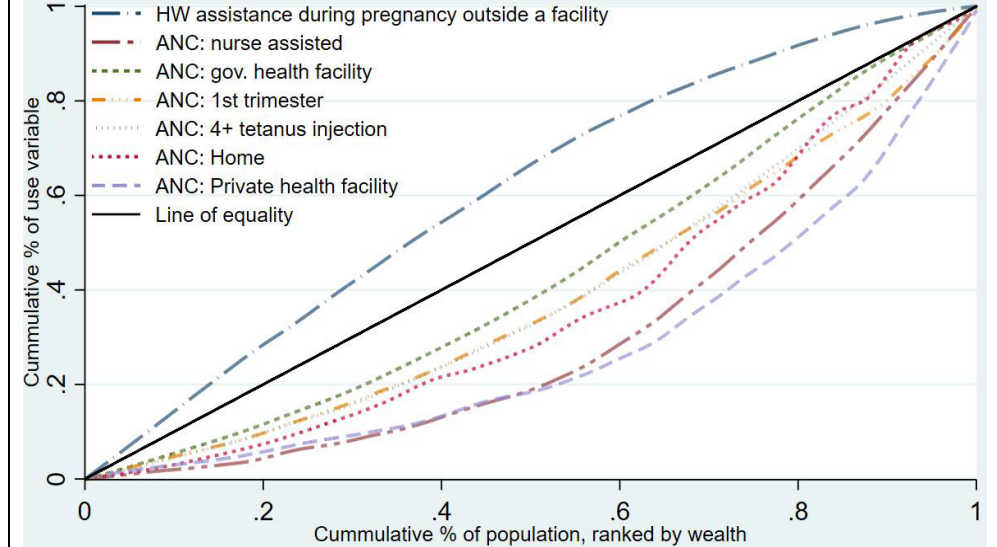

Supplement: Supplementary file 5 — Figure S4. Concentration curves of use of Antenatal care, Nigeria (Years 2003, 2008, 2014). (PDF 1208 kb) [file 12884_2018_2102_MOESM5_ESM.pdf]

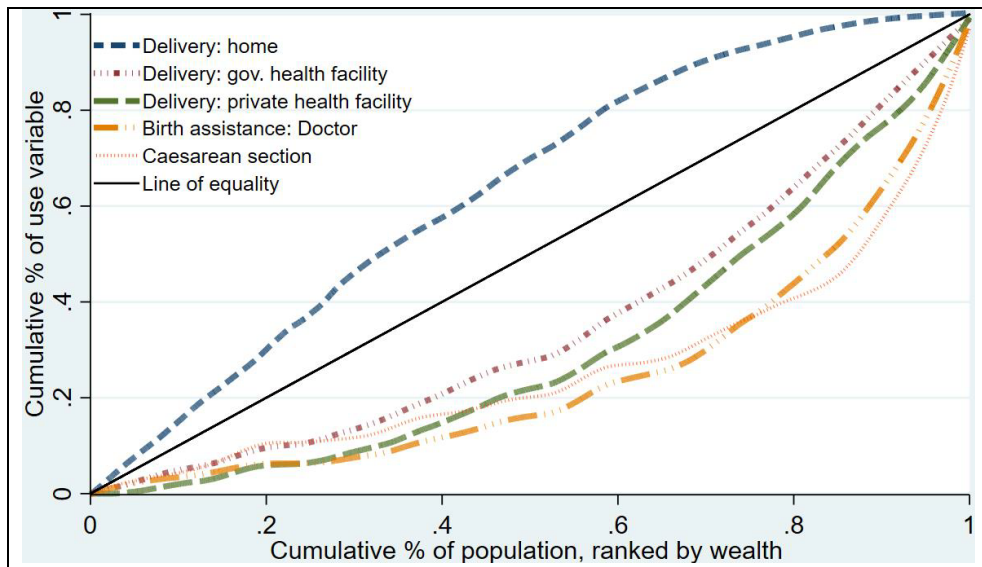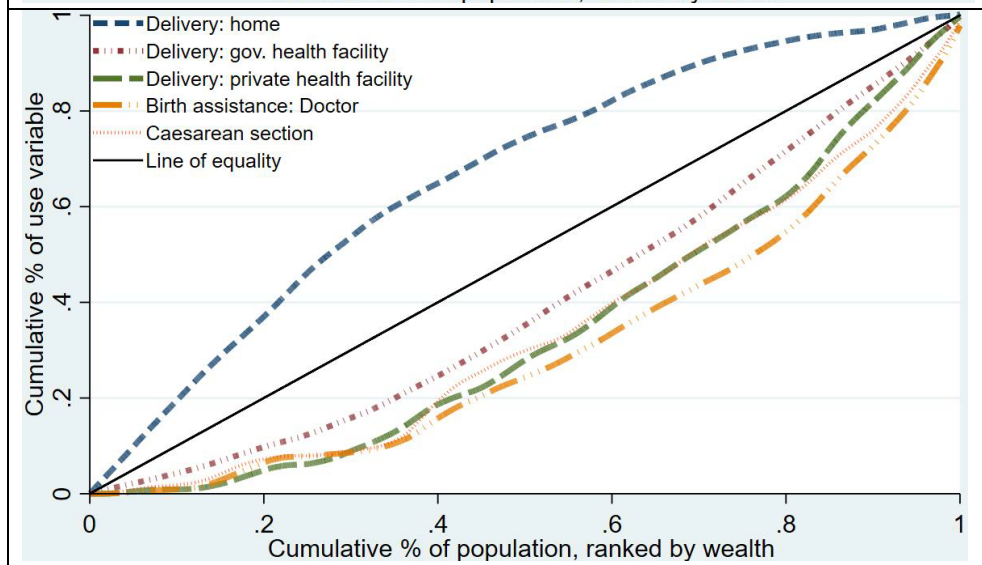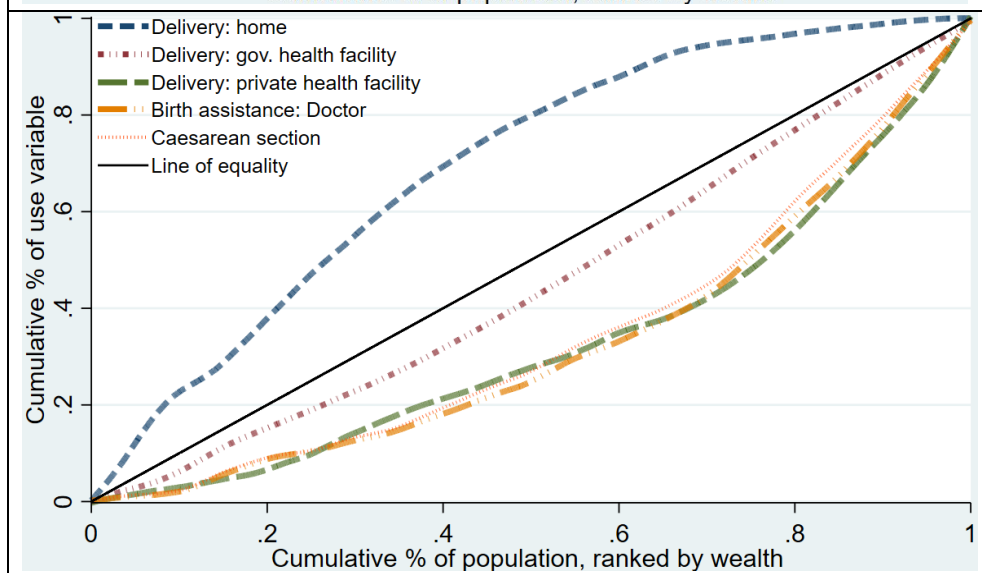

Supplement: Supplementary file 6 — Figure S5. Concentration curves of use of Delivery care, Ghana (Years 2003, 2008, 2014). (PDF 899 kb) [file 12884_2018_2102_MOESM6_ESM.pdf]

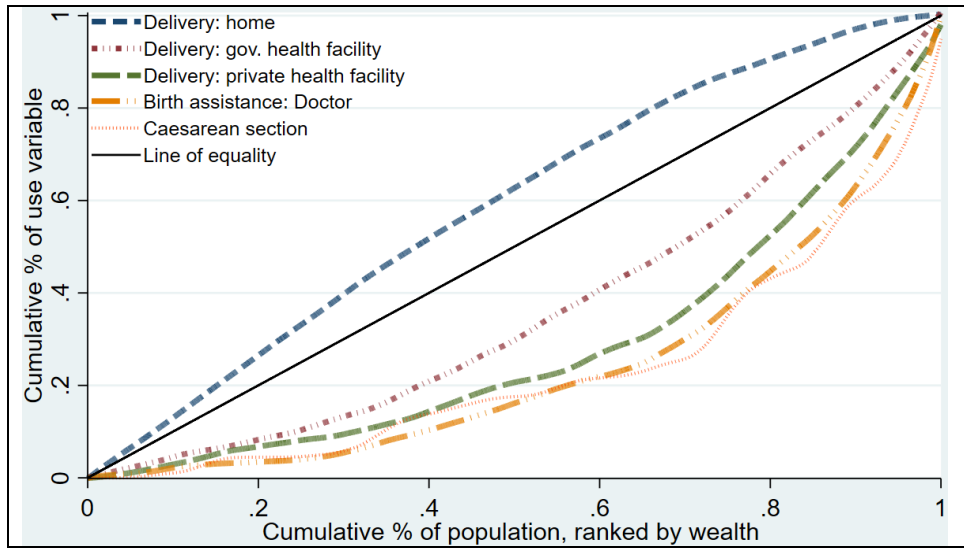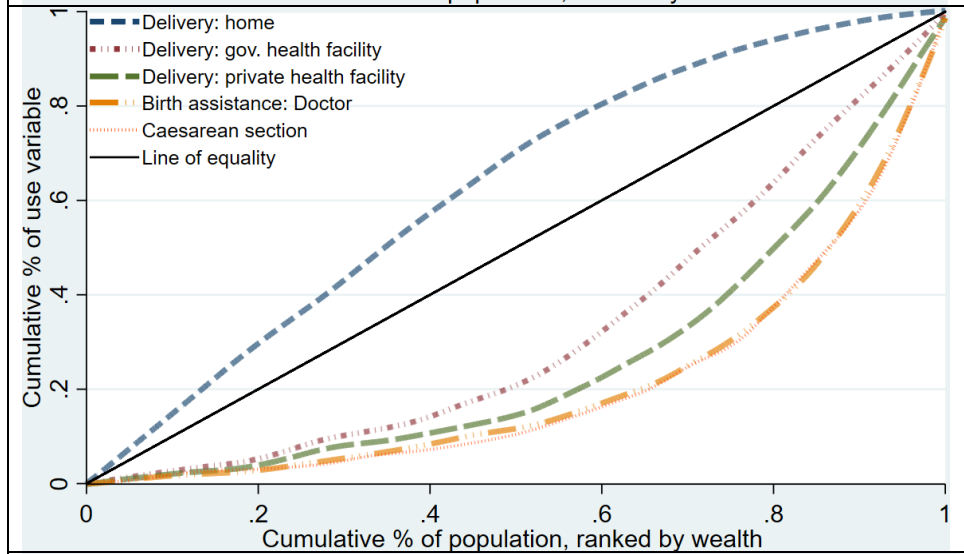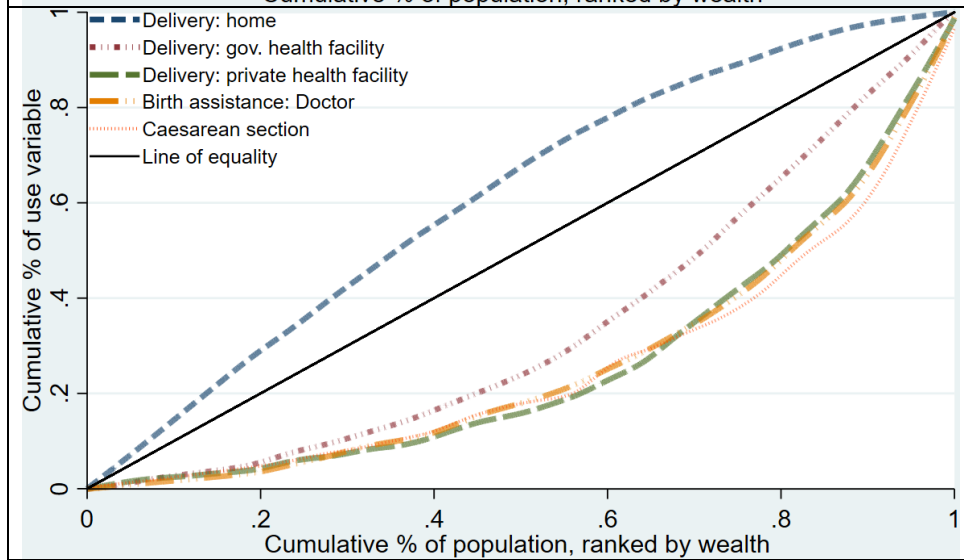

Supplement: Supplementary file 7 — Figure S6. Concentration curves of use of delivery care, Nigeria (Years 2003, 2008, 2013). (PDF 393 kb) [file 12884_2018_2102_MOESM7_ESM.pdf]
